# Long-Range Three-Dimensional Tracking of Nanoparticles Using Interferometric Scattering Microscopy

**DOI:** 10.1021/acsnano.4c08435

**Published:** 2024-10-21

**Authors:** Kiarash Kasaian, Mahdi Mazaheri, Vahid Sandoghdar

**Affiliations:** † Max Planck Institute for the Science of Light, 91058 Erlangen, Germany; ‡ Max-Planck-Zentrum für Physik und Medizin, 91058 Erlangen, Germany; ¶ Department of Physics, Friedrich-Alexander-Universität Erlangen-Nürnberg, 91058 Erlangen, Germany

**Keywords:** interferometric scattering microscopy (iSCAT), interferometry, three-dimensional tracking, single-particle tracking (SPT), diffusion

## Abstract

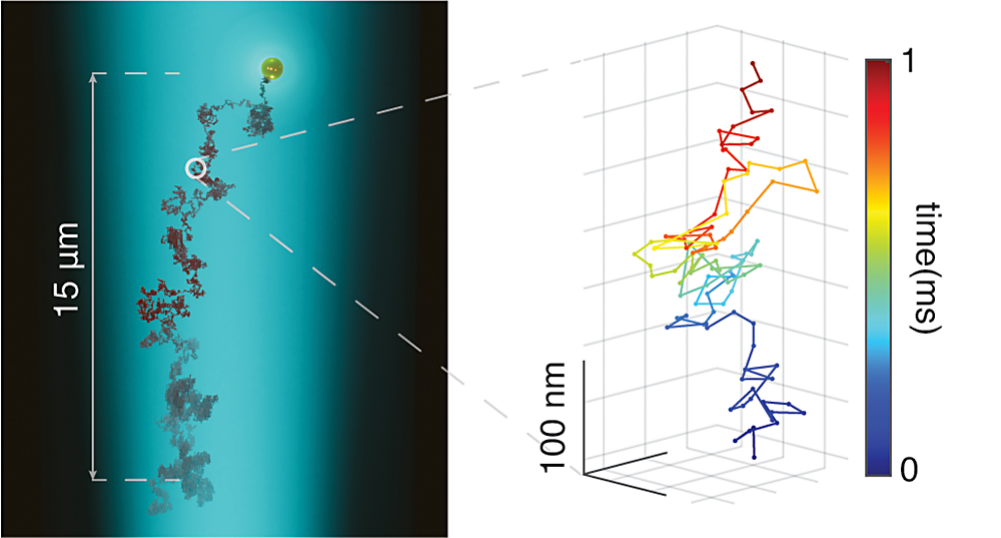

Tracking nanoparticle movement is highly desirable in many scientific areas, and various imaging methods have been employed to achieve this goal. Interferometric scattering (iSCAT) microscopy has been particularly successful in combining very high spatial and temporal resolution for tracking small nanoparticles in all three dimensions. However, previous works have been limited to an axial range of only a few hundred nanometers. Here, we present a robust and efficient measurement and analysis strategy for three-dimensional tracking of nanoparticles at high speed and with nanometer precision. After discussing the principle of our approach using synthetic data, we showcase the performance of the method by tracking gold nanoparticles with diameters ranging from 10 to 80 nm in water, demonstrating an axial tracking range from 4 μm for the smallest particles up to over 30 μm for the larger ones. We point out the limitations and robustness of our system across various noise levels and discuss its promise for applications in cell biology and material science, where the three-dimensional motion of nanoparticles in complex media is of interest.

## Introduction

Single-particle tracking (SPT) is a powerful technique for investigating the dynamic interaction of individual nanoparticles with heterogeneous environments.^1,2^ Over the past three decades, SPT has been extensively applied to studies of diffusion and transport in very different contexts, spanning cell biology and biophysics^3,4^ to material science^5,6^ and statistical physics.^7−9^ The key step in SPT is to image an isolated nano-object onto a well-defined intensity distribution, namely the point-spread function (PSF) of the optical system in use. By fitting a known theoretical or experimental model to the PSF, one can pinpoint the particle’s location in each video frame and establish its trajectory over time. It follows that the localization precision in each frame is dictated by the signal-to-noise ratio (SNR) of the PSF over its background, whereby the signal, background and noise levels depend on various imaging modalities and sample conditions.^10−12^ The available SNR puts a fundamental limit on the size of a nano-object and the speed with which it can be tracked. As an example, large signals from particles such as a micrometer-sized bead used in optical tweezer experiments can yield Ångstrom localization precision within 0.1 s.^13^

The PSF in conventional microscopy techniques such as fluorescence and dark-field scattering is solely based on intensity and can usually be approximated by a Gaussian function. Because in these methods the PSF is more extended in the third dimension, the axial localization precision is lower than in the lateral plane. Furthermore, it becomes increasingly difficult to track particles that move away from the imaging plane. As a result, the great majority of works have only recorded two-dimensional (2D) projections of the 3D particle trajectories. Many methods such as multifocal plane imaging^14−17^ and PSF engineering^18^ have been applied to extend the axial range. A powerful alternative approach for performing high-precision axial tracking is to use interferometric microscopy.^19,20^ Interferometric measurements are particularly advantageous due to the use of phase information along the axial direction, which allows precise monitoring of the motion of a particle away from the imaging plane.^21^ Here, we introduce a robust and efficient technique for 3D nanoparticle tracking at high speed and with nanometer precision. In the following, we explain the principles of our method and demonstrate its effectiveness by tracking gold nanoparticles (GNPs) with diameters ranging from 10 to 80 nm in water.

## Results and Discussion

**Figure 1:**
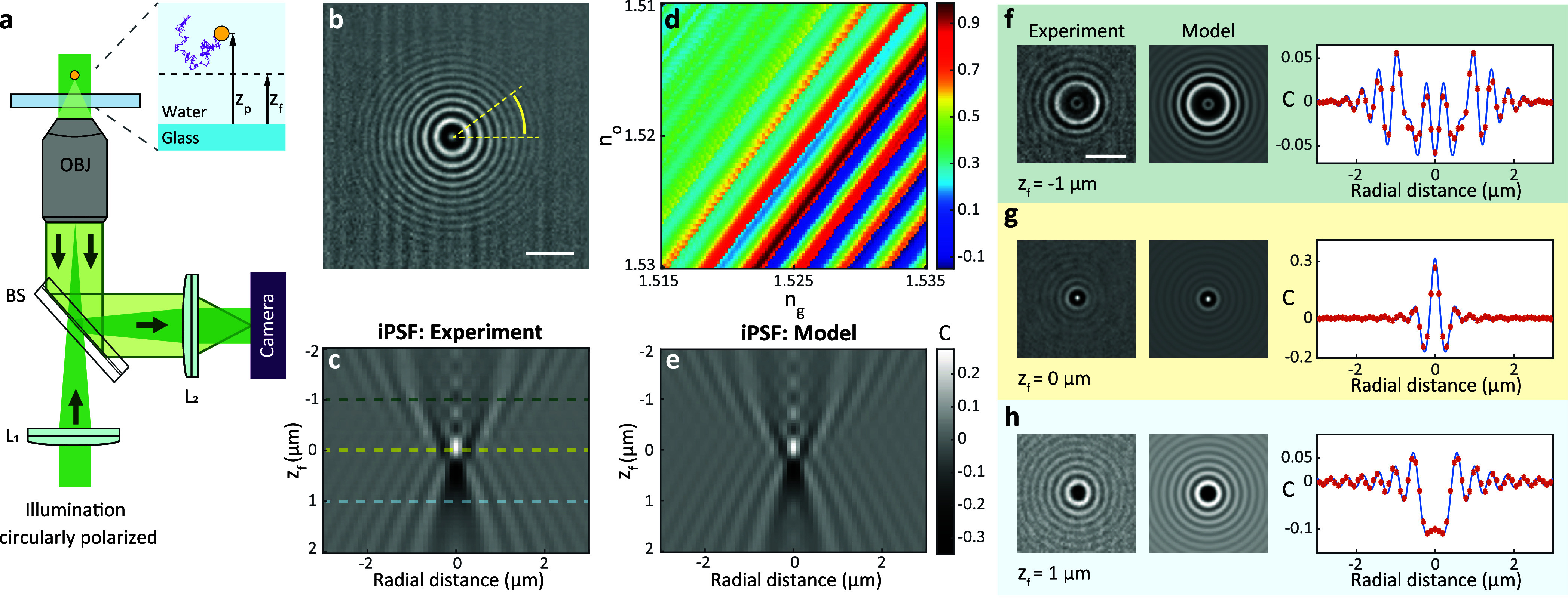
(a) Schematics of a wide-field iSCAT setup. L_1_, BS, OBJ, and L_2_ indicate the wide-field lens (Thorlabs LBF254–200-A), beam splitter (Thorlabs BSW16), microscope objective (Olympus UPLSAPO100XO), and imaging lens (Thorlabs, AC508–500-A), respectively. The inset indicates the focal plane and the axial position of a moving particle (*z*_p_) for a general scenario. In the calibration measurements, *z*_p_ is the radius of the particle. (b) iSCAT image of a 40 nm GNP placed on the cover glass and immersed in water, after temporal median background correction. (c) Measured iPSF stack of the GNP in (b) at different focal plane positions averaged over the azimuthal angle. (d) Normalized correlation values (see color bar) between the measured iPSF and the modeled iPSF with different *n*_g_ and *n*_o_. (e) Calibrated iPSF model based on the optimized values of refractive indices of oil (*n*_o_ = 1.51833) and glass (*n*_g_ = 1.52696) and glass thickness (*t*_g_ = 170 μm). (f–h) Comparison between the measured and modeled iPSF including overlays of their radial profiles for focal plane at *z*_f_ = −1 μm (f), *z*_f_ = 0 μm (g), and *z*_f_ = 1 μm (h). The right-hand plots show the average of the radial profiles computed over all angles for each case. Red symbols represent the experimental data. The blue curves show the respective theoretical models.

Interferometric methods such as holography have been used to track particles in different arrangements, albeit mostly addressing relatively large objects.^20^ In the case of particles much smaller than the wavelength of light, the optical response is governed by Rayleigh scattering, and the method of choice is referred to as interferometric scattering microscopy (iSCAT).^22,23^ This method exploits a homodyne detection scheme, where the scattered electric field from a nano-object interferes with the field of a reference beam. In the wide-field mode (see Figure 1a), a nearly collimated illumination is realized by focusing a light beam at the back focal plane of the microscope objective. Technical details of iSCAT microscopy can be found in previous publications.^23,24^

In the most common form of iSCAT, the reference beam is formed from the reflection of the illumination beam at the interface between the sample medium and the substrate supporting it. The detected iSCAT signal in this arrangement can be written as
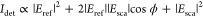
1where the reference field *E*_ref_ = *rE*_inc_ is reflected from the medium-glass interface, *E*_sca_ = *sE*_inc_ represents the light scattered from the sample, and ϕ is the phase difference between *E*_ref_ and *E*_sca_. To account for potential variations in the illumination beam, we normalize the iSCAT images to *I*_ref_ = |*E*_ref_|^2^ and define the contrast *C* as
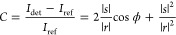
2

Over the past two decades, many efforts have demonstrated the high sensitivity of iSCAT for detection of nanoparticles down to single viruses^25^ and even single small proteins.^26−28^ As compared to fluorescence SPT, single-particle tracking based on iSCAT (iSPT) has a nearly infinite photon budget because it suffers neither from saturation nor from photobleaching. Therefore, iSPT provides access to both high temporal resolution and long-duration measurements.^19,25,29,30^ Another decisive advantage of iSCAT is that its interferometric nature makes the signal highly sensitive to the axial position of the nanoparticle under study.^31−36^ However, the ambiguity resulting from the periodicity of the modulating traveling phase prevents one from determining the axial direction of travel over a range longer than about λ/4, where λ is the wavelength of light in the medium of interest. To get around this problem, one can exploit the axial asymmetry of the spherical aberration about the focal plane. It, thus, follows that the radial cross-section of the interferometric point-spread function (iPSF) contains information about the height of the particle above the cover glass.^37,38^

In our previous efforts, we exploited the axial asymmetry caused by spherical aberration combined with a machine learning scheme to reach an axial tracking range of approximately 300 nm.^19,37^ However, an extension of this approach to longer axial ranges was challenging due to the limitations of the unsupervised machine learning scheme based on *k*-means clustering.^39^ These limitations include increased computational complexity and potential inaccuracies in clustering as it relies on the silhouette values for determining the optimal number of clusters. In our current work, we establish a suitable estimator to assign the full lateral content of the experimental iPSF along a trajectory to the computed iPSFs specific to our iSCAT setup. To achieve this, we calibrate the imaging system carefully and establish a computational workflow to model the experimental iPSF.

### 3D-iSPT Algorithm

To establish an accurate 3D model of the iPSF for a given optical setup (see Figure 1a), we first measured the iPSF profile of single nanoparticles at the water–glass interface as the focal plane of the microscope objective was scanned through a range of 4 μm. Here, we used circularly polarized light to average over all the induced dipole orientations, yielding iPSFs with circular symmetry (see Figure 1b). Thus, we averaged the radial profile of the iPSF over the azimuthal angle at each focal plane and used the outcome as a representation. An example of a radial iPSF stack of a 40 nm GNP is depicted in Figure 1c.

To optimize the model, we maximized the Pearson correlation value between the experimental and modeled iPSF stacks (see Supporting Information (SI) eq S1), considering different setup parameters such as the thickness of the cover glass (*t*_g_) as well as the refractive indices of the immersion oil (*n*_o_) and glass (*n*_g_). Figure 1d illustrates an example of the correlation value optimization process as a function of *n*_o_ and *n*_g_. The diagonal trend accounts for the compensation of the accumulated phase in the glass substrate and immersion oil, leading to similar iPSF stacks (see Figure S2). This is because the total phase accumulated in the glass and oil layers is more important than the phase accumulated within each layer separately, particularly within the range of refractive indices shown in Figure 1d. Figure 1e exhibits the corresponding modeled iPSF stack for the GNP under study, demonstrating an agreement with 99% correlation with the experimental measurements for *n*_o_ = 1.51833 and *n*_g_ = 1.52696. To highlight the asymmetry of the iPSF relative to the focal plane, we depict the experimental and modeled iPSF images and their radial profiles for *z*_f_ = −1, 0, and 1 μm in Figure 1f–h, respectively.

**Figure 2:**
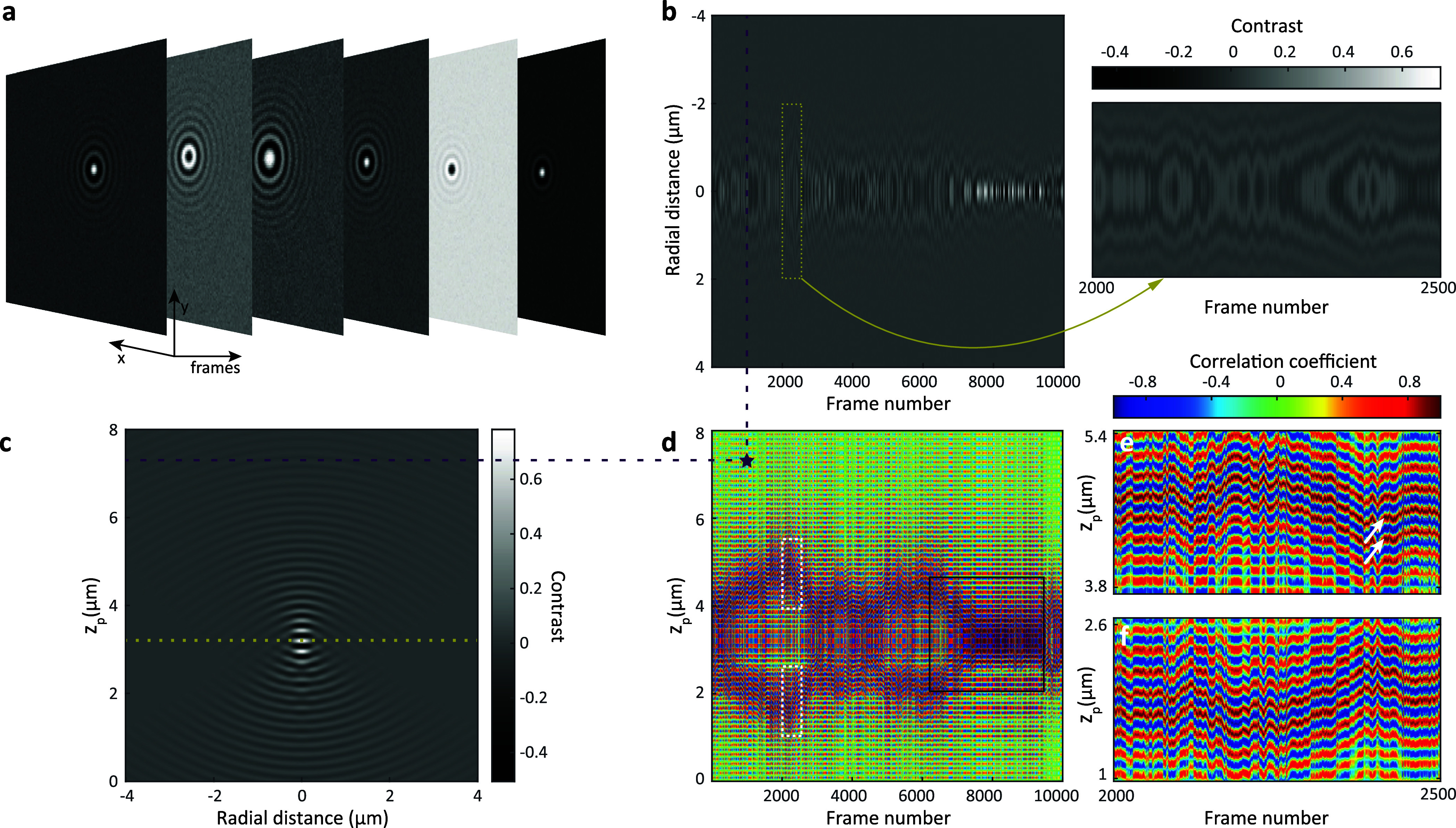
(a) Synthetic iSCAT images of a 40 nm GNP diffusing in water in 3D considering shot noise based on the camera electron well capacity (σ_*n*_ = 6.56 × 10^–3^). (b) Radial iPSF cross-section of the GNP throughout its trajectory. The inset shows a close-up view of the region marked by the yellow rectangle. (c) Calculated iPSF model of a 40 nm GNP for the focal plane set at 3.2 μm above the cover glass (indicated by the dotted yellow line). (d) Correlation map of the trajectory. The star indicates the correlation coefficient between the extracted iPSF profiles from synthetic data and modeled iPSF profiles indicated by dashed lines in (b) and (c). Black square marks the region where the stripes from both sides of the focal plane join as *z*_p_ approaches *z*_f_. (e) Close-up view of the correlation map within the upper white dashed rectangle in (d), highlighting the striped patterns above the focal plane (*z*_p_ > *z*_f_). (f) Close-up of a similar section (lower dashed rectangle in (d)) shows the details beneath the focal plane (*z*_p_ < *z*_f_), revealing the partial mirror symmetry of the stripes in relation to the focal plane. (d)–(f) share the same color bar, representing normalized correlation values.

Next, we computed synthetic trajectories for a GNP that experienced Brownian motion in water (see Supporting Information: synthetic videos). The random step sizes of such a particle follow a normal distribution with a standard deviation of , where *D* is the particle’s diffusion coefficient in the medium, and Δ*t* is the time between two consecutive frames. We reconstructed images at a frame rate of 100 kHz to match our experimental acquisition rate acquired by a high-speed camera (Phantom V1610) with a full-well capacity of 23,200 electrons (see Figure 2a). A shot noise with a standard deviation of σ_*n*_ = 6.56 × 10^–3^ is added to the synthetic images to simulate the experimental shot noise.

To localize a particle in the lateral plane, we applied radial variance transform (RVT)^40^ to the iPSF in each frame. This information was used to generate a cross-section map (see Figure 2b), representing the evolution of the radial iPSF profile over time. We then employed a normalized correlation map in order to compare the temporal evolution of the experimental radial profiles to those of the model. The normalized correlation map is calculated as
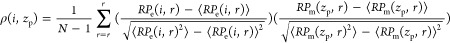
3where radial profiles *RP*_e_(*i*, *r*) and *RP*_m_(*z*_p_, *r*) represent the radial cross sections of the experimental and modeled iPSF, respectively. *RP*_e/m_(*i*, *r*) is a function of the frame index (*i*) and of the radial distance (*r*), where *r* takes on discrete values of *r*_1_ to *r*_N_ and denotes the distance from the center of the iPSF to a given pixel. The parameter *z*_p_ denotes the axial position of the particle, and ⟨·⟩ represents averaging over *r*. The dashed lines in Figure 2b,c (representing the radial profiles) and the star in Figure 2d (corresponding correlation value) show examples, where the correlation value of the model with the radial profile from each simulated frame is calculated at various *z*_p_.

Figure 2d shows the resulting correlation map ρ(*i*, *z*_p_). Figure 2e,f displays a close-up of two regions marked in (d). The phase difference caused by the extra travel between the cover glass and the particle leads to the modulation of the correlation map along *z*_p_ so that the normalized correlation value at a particular frame oscillates rapidly with a periodicity of . This results in the fluctuation of the correlation between negative and positive values along the vertical axis, giving rise to a striped pattern. If the frame rate is sufficiently high such that the particle’s axial displacement between two consecutive frames does not exceed , these areas will be linked to one of their neighboring frames on the correlation map.

We remark that a direct assignment of the axial location to the highest correlation values in a frame-by-frame procedure may lead to erroneous results, as various noise factors and setup imperfections can give rise to three potential scenarios: (1) The maximum correlation value within a given frame might not correctly pinpoint the true axial position among different stripes in the presence of noise. The white arrows in Figure 2e show an example of two very close correlation values of 0.99 and 0.98 in two neighboring stripes. (2) The extracted axial position of the particle undergoes jumps between the stripes situated above and below the focal plane (shown in Figures 2e,f, and S4). (3) When the particle is near the focal plane the iPSF exhibits the least spatial features, as the scattered light is mostly concentrated in the center of the iPSF. Hence, the difference between the correlation value corresponding to the true *z*_p_ and its respective mirror on the other side of the focal plane becomes minimal (see Figure S4). Consequently, using the maximum correlation at each frame yields inaccurate localization. Furthermore, as highlighted in the dark square in Figure 2d, axial tracking becomes even more challenging when a particle repeatedly crosses the focal plane along its trajectory. To overcome these challenges, we implement an algorithm that leverages the full spatiotemporal properties of the correlation map, thus, using the information on all the frames for determining the particle’s axial location. Our algorithm also utilizes a graph representation of the correlation map to determine the axial position when the particle diffuses near the focal plane.

**Figure 3:**
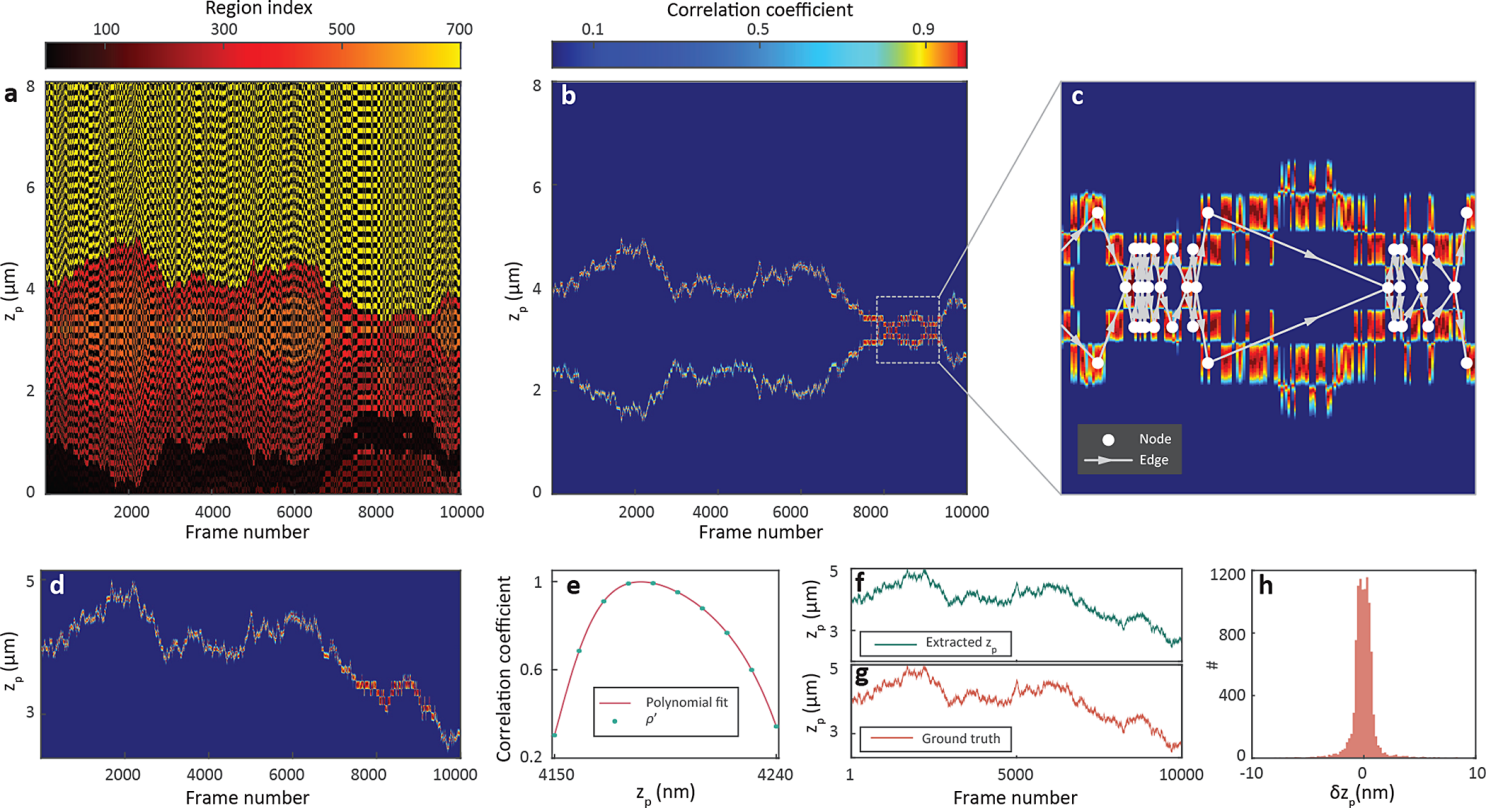
(a) Labeled correlation map of Figure 2d. Color bar shows the integer numbers assigned to each region. (b) Selected region on the correlation map with the highest correlation score. Color bar represents the correlation coefficients. (c) Magnified view of a selected region marked by the white dashed box in (b), where the particle crosses the focal plane multiple times. Lines and circles indicate the edges and nodes of the branching graph. (d) Debranched selected region of (b). (e) Example of adaptive polynomial fitting for finding the location of the maximum correlation value for a frame. (f) Extracted values of the axial positions. (g) Ground truth of the axial positions. (h) Histogram of the axial localization error of (f) with a standard deviation of 1.46 nm.

We start by finding the region with the highest total correlation value. To achieve that, we first convert the correlation map to a binary image by setting a global threshold at zero. Next, we segment and isolate connected regions within this binary map that have values of 1 (see Figure S5). This results in various regions *R*_*j*_, which we label with unique integer numbers. Figure 3a illustrates more than 900 regions that arise from the correlation map in Figure 2d. Each region *R*_*j*_ is then assigned a score *S*_*R*_*j*__ calculated as the sum of the maximum correlation coefficients over all its frames,
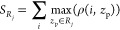
4The region with the highest score, denoted as *R*_max_, is selected for further analysis. Then the binary mask of *R*_max_ is multiplied with the original correlation map to obtain a new map that exclusively contains the values corresponding to *R*_max_. Figure 3b shows that this procedure results in a clear selection of a region (ρ′(*i*, *z*_p_)) with high correlation values. The two branches within the beginning of the selected region above and below the focal planes can be avoided if one adjusts the focal plane to the cover glass interface or sets it well above the tracking region. We choose to place the focal plane roughly in the middle of the volume of interest because this increases the tracking range.

Branching within the selected region in Figure 3b prevents one from determining a maximum correlation value for the axial localization. If the local maximum values associated with two or more branches within the selected region are close to each other, frame-wise assignment of the maximum correlation can experience false jumps in the 3D trajectory, leading to inaccurate localization. To overcome this hurdle, we identify the branching points (the frames at which the number of branches varies) and create a directional graph to represent these and the sections in ρ′(*i*, *z*_p_). Figure 3c depicts the correlation map near the focus and its corresponding graph representation, illustrating occurrences of multiple branching (or complex branching) within the selected region as the particle diffuses near the focal plane.

We divide the selected region into sections in which the number of branches remains constant. The nodes and edges of the graph represent branching points and sections in ρ′(*i*, *z*_p_), respectively. The directionality of the edges in the graph signifies the chronological order of the frames in the trajectory, ensuring that the paths progress forward in time. An example of the directional graph is presented in Figure 3c. Every edge of the graph has a distance
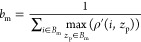
5where *m* is the index of the branch *B*_m_. Distances *b*_m_ calculated by eq 5 are inversely related to the correlation values. Thus, the path with the minimum distance in the graph includes the largest sum of the correlation values along the trajectory. For a given source node, Dijkstra’s algorithm^41^ can find the shortest path between any two nodes in the graph with an optimized computational overhead. Following this procedure, the final single-branch correlation map is reconstructed from the shortest path of the graph, which is depicted in Figure 3d. We remark that computing the large number of possible combinations would present a daunting challenge. For instance, if the selected region toggles 23 times between one and two branches, the number of alternative paths from the first to the last frame amounts to 2^23^ (≈8 × 10^6^). A brute-force approach to determining the optimal path is, thus, not viable for long trajectories.

To refine the axial localization beyond the axial discretization of the modeled iPSF, we fit an adaptive polynomial function to the correlation values at each frame, whereby the degree of the polynomial depends on the sampling rate of the iPSF along *z*_p_ (1 nm in this example; see Figure 3e). The maximum of the fitted polynomial allows us to extract the particle’s axial position along the trajectory. As seen in Figure 3f–h, the algorithm accurately localizes the axial position when compared to the ground truth. In a representative example, using a standard desktop computer with a hexa-core processor and 64GB RAM, a trajectory of 10,000 frames with 48 nodes and 73 branches (3.35 × 10^7^ possible outcomes) was processed in approximately 25 s. This demonstrates the practicality of our method for real-world applications without excessive processing time.

### Localization Error and Robustness

We now assess our algorithm’s performance under various conditions. The axial localization error (δ*z*_p_) depends on *z*_f_, *z*_p_, shot noise, and lateral localization error (δ*r*_p_). The geometry of the iPSF is influenced by both *z*_f_ and *z*_p_, which in turn impacts δ*z*_p_. Shot noise and lateral localization precision both affect the extracted radial profile, the former by modifying the average over azimuthal angles and the latter by introducing an error in the identification of the iPSF’s center of symmetry.

**Figure 4:**
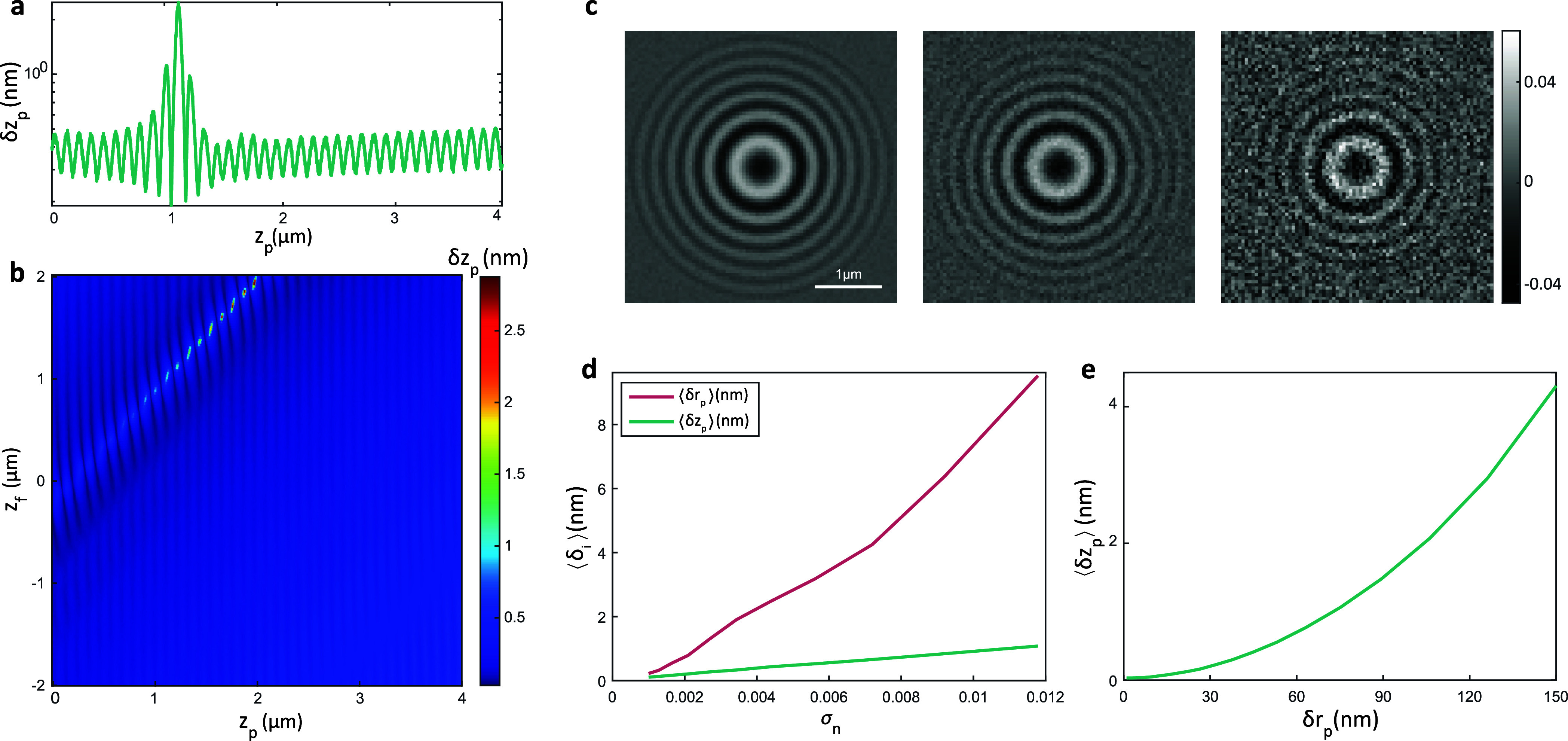
(a) Axial localization error as a function of the true axial position of a 40 nm GNP for *z*_f_ = 1 μm based on synthetic data. (b) Standard deviation of the error (δ_*z*_(nm)) in estimating the axial position of a 40 nm GNP due to the presence of noise for different focal planes and particle axial distances. In (a, b), the normalized shot noise level is σ_*n*_ = 6.56 × 10^–3^. (c) Exemplary synthetic iSCAT images of a 40 nm GNP in water at 5.5 μm above the cover glass with focal plane set at 3.2 μm with different shot noise levels. From left to right: the normalized standard deviation of shot noise is 1 × 10^–3^, 3.43 × 10^–3^, and 1.18 × 10^–2^, respectively. The color bar represents the iSCAT contrast. (d) Lateral and axial localization uncertainty as a function of shot noise standard deviation calculated for a 40 nm GNP in water. The uncertainty is averaged over 8 μm axial range with the focal plane at 3.2 μm above the cover glass. (e) Calculated axial localization uncertainty as a function of the lateral position error for the same axial range and focal plane position as in (d), when σ_*n*_ = 0.

First, we assess how *z*_f_ and *z*_p_ influence δ*z*_p_. Here, we assume a particle is laterally fixed (i.e., δ*r*_p_ = 0) and consider a fixed level of shot noise, anticipated from our measurements. For every *z*_f_ and *z*_p_, we generate a large number (1000) of random realizations of the shot noise and apply our localization algorithm to the resulting noisy iPSFs to localize the particle. The axial localization error for every *z*_p_ and for a given *z*_f_ is then calculated by measuring the standard deviation of the differences between the retrieved positions and their known axial locations. Figure 4a shows the resulting axial localization error for a 40 nm GNP as a function of *z*_p_. The axial range spans [0, 4] μm when *z*_f_ = 1 μm above the cover glass interface. This procedure is repeated for a series of focal planes in the range *z*_f_ = [ – 2, 2] μm. Figure 4b displays the resulting δ_*z*_ as a function of *z*_f_ and *z*_p_. As the particle approaches the focal plane, its iPSF exhibits fewer radial features, leading to an increase in axial uncertainty. Nevertheless, the highest error in estimating the axial position remains at only a few nanometers. The mean localization error within *z*_p_ = [0, 4] μm and *z*_f_ = [−2, 2] μm amounts to 0.2 nm.

We also evaluated the impact of the shot noise on δ*z*_p_ and δ*r*_p_ by generating iSCAT images of a 40 nm GNP in water. Figure 4c illustrates the iPSF at different shot noise levels. We simulated videos for a particle undergoing linear axial movement in the range *z*_p_ = [0, 8] μm with *z*_f_ = 3.2 μm above the cover glass. We then applied our 3D tracking algorithm, whereby δ*z*_p_ and δ*r*_p_ were averaged over *z*_p_ for each noise level, ranging from σ_*n*_ = 1 × 10^–3^ to 1.2 × 10^–2^. Here, we define the normalized shot noise level as  with *N*_e_ representing the average number of electrons per camera pixel. As shown in Figure 4d, our axial localization algorithm achieves higher precision compared to the lateral localization using the state-of-the-art RVT method. This is in agreement with the predictions of a Cramér–Rao lower bound analysis for localization in the axial direction in iSCAT microscopy.^42^

As previously stated, our algorithm operates on the radial profiles that are obtained after performing the lateral localization. To examine the sensitivity of the algorithm to the lateral localization error, we set a range of offsets for the lateral localization in the interval [0, 150] nm, which is close to the diffraction limit in our setup. In this analysis, we did not add shot noise to the data. In Figure 4e, we present the average axial localization error ⟨δ*z*_p_⟩ over the range *z*_p_ = [0, 8] μm as a function of the lateral offset. This average error is calculated by first determining the absolute error in the axial localization at each *z*_p_ and then computing the mean of these absolute errors across different axial positions. We find that ⟨δ*z*_p_⟩ remains below 5 nm even with a lateral localization offset of 150 nm.

When considering stationary particles, an extended integration time allows for more photon collection, which reduces the effect of shot noise and enhances the signal-to-noise ratio. Consequently, as depicted in Figure 4d, longer integration times (lower σ_*n*_) provide higher lateral and axial localization precisions. However, when dealing with moving nanoparticles, the integration time must be chosen carefully to avoid motion blurring.

### Experimental Results

We now present an experimental demonstration of 3D tracking applied to GNPs of different sizes diffusing in water. Capturing the trajectories of such nanoparticles across a large axial range without interruption is challenging because the particle is likely to leave the field of view (FOV) laterally. In more sophisticated experiments, a feedback loop can be implemented to compensate for this phenomenon. Here, we addressed the issue by recording several videos of different particles for each size category around different axial positions, while keeping the focal plane fixed. By analyzing these videos, we estimated the ranges over which our algorithm can effectively track. We note, however, that the tracking range eventually depends on the strength of the iSCAT signal. As a result, larger particles can be tracked over a larger axial range.

In our measurements, we used a cover glass (Schott D 263) with a thickness of 170 μm and created a liquid chamber by placing a gasket on top (CoverWell). Then, we added 110 μL of DI water and 7 μL of a suspension containing GNPs. In order to ensure mechanical stability, the sample rested for approximately 15 min. To control and assess the position of the focal plane, we marked the upper surface of the cover glass with spin-coated GNPs (diameter 80 nm) prior to the chamber assembly. The axial position of the cover glass was calibrated relative to the maximum-bright central contrast of the 80 nm GNPs. After localizing the cover glass surface, we used the calibration of the piezo-electric scanner to displace the sample by a precise amount.

**Figure 5:**
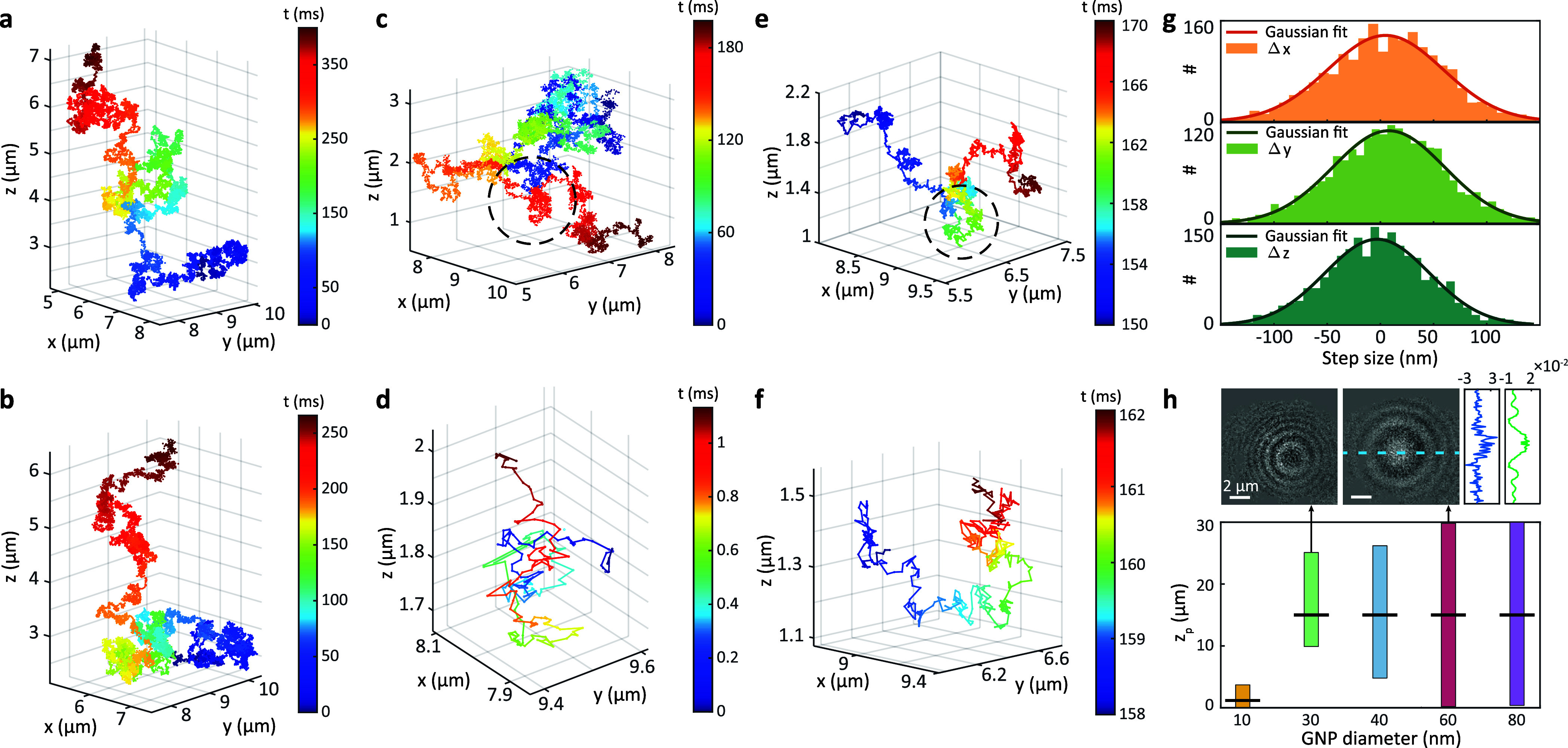
Exemplary experimental 3D trajectories of GNPs with diameters of 80 nm (a), 60 nm (b), 40 nm (c), and 10 nm (d) diffusing in water. Trajectories shorter than 1000 frames were excluded from the analysis. For 10 nm GNPs, however, we chose a lower threshold of 200 frames. Color bars represent time. (e) Close-up of the trajectory is shown in (c). (f) Enlarged view of the marked region in (e). (g) Step size distributions along all axes in (e), with 100 μs time interval, fitted with Gaussian functions. The standard deviations of the fitted Gaussians are σ_*x*_ = 54 nm, σ_*y*_ = 52.6 nm, and σ_*z*_ = 49.1 nm. (h) Axial ranges of the GNPs of different sizes tracked in our measurements. Solid black horizontal lines represent the focal plane for the respective measurements. Top-left and top-right inset images show 30 and 60 nm GNPs at 25.1 and 29.9 μm above the glass–water interface, respectively. A line-cut (blue) and the angular average of several such cuts (green) from the image of the 60 nm GNP are shown in its margin.

Videos of 60, 40 and 30 nm GNPs were recorded at a frame rate of 100 kHz. For 80 and 10 nm GNPs, we used 70 and 200 kHz frame rates, respectively. These videos covered a FOV of 128 × 128 pixels corresponding to an area of 13 μm × 13 μm on the sample. The laser (Toptica iBEAM SMART 515) power was adjusted so that the background level was near saturation. For larger GNPs (80 and 60 nm), the laser power was set such that the reflection filled approximately half of the camera’s full electron well capacity. This adjustment was made to prevent saturation when these larger GNPs diffuse near the focal plane. To compensate for laser power fluctuations, we normalized the pixel values of each frame to the sum of the pixel values of that frame. Then the background was subtracted using temporal median background correction, and RVT was applied to extract radial profiles in each frame. In the final step, the axial localization algorithm was applied. Figure 5a–d shows examples of 3D trajectories from freely diffusing GNPs of different sizes.

Figure 5e provides a close-up view of the trajectory within the dotted circle marked in Figure 5c,f displays a further close-up view of the trajectory within the dotted circle marked in Figure 5e. Figure 5g depicts the step size distributions in 100 μs time interval along the three axes for the trajectory in Figure 5e, confirming the isotropic Brownian motion. By fitting a line to the mean square displacement (MSD) plots of the 3D trajectories,^43^ we extract the mean value of the diffusion coefficients (⟨*D*_3D_⟩). For 10 nm GNPs in water at 297 K, we obtain ⟨*D*_3D_⟩ = 43.7 ± 1 μm^2^/s, which agrees with the theoretical prediction of *D* = 44.1 μm^2^/s. MSD plots for the tracked particles along the *x*, *y*, and *z* axes are shown in Figures S16 and S17. It is worth noting that due to motion blurring, the axial localization error estimated using the offset of the MSD curve^43^ is larger than the theoretical limitations presented in Figure 4. For instance, the displacement of a 40 nm GNP during an exposure time of 10 μs follows a Gaussian distribution with a standard deviation of 15.5 nm in each direction, limiting its localization accuracy (see Supporting Information).

To demonstrate the long axial tracking range capability of algorithm, we conducted additional measurements with the focal plane set at 15 μm above the interface for GNP sizes of 80, 60, 40, and 30 nm (see Figure S11). In Figure 5h, we present the axial ranges of the GNPs, measured in our experiments, along with the iSCAT images for the maximum and minimum axial positions for 60 and 30 nm GNPs. The measured axial ranges for 80, 60, 40, 30, and 10 nm GNPs were around 30, 30, 22, 15, and 3.7 μm, respectively. This was achieved without scanning the focal plane or employing methods such as bifocal imaging and PSF engineering. These results demonstrate that iSCAT provides adequate signal for detecting and tracking particles, even when the iPSFs are highly defocused. Although our algorithm is designed to work optimally with sparsely distributed particles in the FOV, it can also handle cases where highly defocused iPSFs overlap. This is because radial profiles are accurately extracted by averaging pixels at similar radii (see Figure S15).

## Conclusions

In most single-particle tracking applications, it is highly advantageous to use very small probes in order to avoid perturbations due to the finite size of the probe. Over the years, particle sizes ranging from several 100 nm down to single quantum dots or molecules have been used, albeit with varying levels of performance in terms of localization precision, accuracy and speed. In this work, we presented an experimental and algorithmic pipeline for high-precision 3D tracking of individual nanoparticles over a large range of tens of micrometers in the axial direction and at a temporal resolution as high as 5 μs. Our approach exploits the full information in the iSCAT point-spread function to establish a correlation between the experimental and model radial profiles in each frame of a video. By employing graph theory and Dijkstra’s algorithm, we differentiate the particle’s position above and below the focal plane in the temporal map of the correlation coefficients.

We applied our algorithm to experimental measurements on nanoparticles with diameters ranging from 10 to 80 nm while they diffused in water. We successfully extracted the 3D trajectories of individual nanoparticles over an extended axial range, reaching at least 30 μm for the larger particles with nanometer localization precisions. We have demonstrated, by simulations and experiments, that iSCAT offers better localization precision in the axial direction than in the lateral dimensions. Moreover, the experimentally determined localization errors are governed by practical conditions such as imaging speed and not by our 3D-iSPT algorithm. Our work presents a superb spatiotemporal precision in 3D tracking of small nanoparticles in highly diffusive liquids, with an extended axial range well beyond the microscope objective’s depth of field. This approach may also find use in holographic techniques.

3D SPT of very small nanoparticles holds great promise in studies of diffusion and transport phenomena in many areas of science and technology. Potential directions include tracking nano-objects within cells and porous membranes, where understanding the dynamics and interactions at the nanoscale is crucial. Our method can also be employed to investigate molecular chemistry, for instance, the interaction between conjugated nanoparticles and functionalized surfaces.

## Methods

### Sample Preparation

High-precision Schott D263 cover glasses with 170 μm thickness were cleaned by submerging them in a 2% Hellmanex III solution and sonicating them for 15 min in an ultrasonic bath. After rinsing with Milli-Q water, the beaker was refilled with Milli-Q water and sonicated again to remove any remaining Hellmanex III. This process was repeated twice, and then the cover glasses were dried using a nitrogen stream. Next, the substrates were plasma-cleaned in oxygen plasma at 500 W for 10 min. After plasma cleaning, 35 μL of a diluted (1:100) 80 nm GNP solution (BBI, batch no. 21040063) was spin-coated onto the cover glass at 3000 rpm for 30 s. A precleaned CoverWell gasket, cleaned via sonication in 2% Hellmanex III and Milli-Q water, was attached to form a chamber. The assembly was mounted on the iSCAT setup, and the focus was adjusted by maximizing the brightness of the central pixel corresponding to the GNPs. Finally, 110 μL of deionized water was added to the chamber, followed by the GNP. For the substrates used for tracking 10 nm GNPs, 35 μL of a diluted (1:10) 40 nm GNP (BBI, batch no. 21040116) was spin-coated onto the cover glass at 3000 rpm for 30 s.

## Supporting Information Available

3D particle tracking code with demonstration (iPSF model; radial profiles; correlation map; and iSCAT video) (ZIP)
Pearson correlation coefficient; synthetic videos; impact of the design values of the microscope objective on iPSF; effect of the refractive index of the immersion oil on iPSF; effect of the particle’s position on the shape of the correlation map; boundary tracing of the correlation map; conversion of the correlation map into directional graph; demonstrating long axial range using synthetic videos; long axial range tracking using truncated iPSFs; methods to track 10 nm diffusing in water; tracking multiple particles in one field of view; mean square displacement of the tracked particles; and list of the nanoparticles used in the experiments (PDF)

## Notes

A preliminary version of this article was published as a preprint.: K.K; M.M.; V.S., Long-range three-dimensional tracking of nanoparticles using interferometric scattering (iSCAT) microscopy. 2024, 2401.12939. arXiv. 10.48550/arXiv.2401.12939 (accessed September 24, 2024).
