# Supplementary material for: Long-Range Three-Dimensional Tracking of Nanoparticles Using Interferometric Scattering Microscopy
Source: ACS Nano. 2024 Oct 21;18(44):30463–72. doi: 10.1021/acsnano.4c08435 (PMC11544927; doi:10.1021/acsnano.4c08435)
Supplement: Supplementary file 2 — nn4c08435_si_002.pdf [file nn4c08435_si_002.pdf]

Supplementary Information: Long-range three-dimensional tracking of nanoparticles using interferometric scattering microscopy

Kiarash Kasaian,[†,‡,¶,§] Mahdi Mazaheri,[†,‡,¶,§] and Vahid Sandoghdar[*,†,‡,¶]

†*Max Planck Institute for the Science of Light, 91058 Erlangen, Germany.*

‡*Max-Planck-Zentrum für Physik und Medizin, 91058 Erlangen, Germany.*

¶*Department of Physics, Friedrich-Alexander-Universität Erlangen-Nürnberg, 91058 Erlangen, Germany.*

§*These authors contributed equally.*

E-mail: vahid.sandoghdar@mpl.mpg.de

Contents

1 Pearson correlation coefficient **3**

2 Synthetic videos **4**

3 Impact of design values of microscope objective on iPSF **6**

4 Impact of immersion oil refractive index on focal plane asymmetry in iPSF **8**

5 Effect of particle position on correlation map **10**

6 Boundary tracing of correlation map **12**

7 Graph representation of the correlation map **14**

8 Extended tracking capabilities across the focal plane **16**

9 Long axial range 3D tracking on synthetic videos **17**

10 Long axial range 3D tracking using truncated iPSF profiles **19**

11 Determining the axial range experimentally **21**

12 Tracking GNPs with signal near noise level **25**

13 Tracking multiple nanoparticles in the iSCAT field of view **28**

14 Mean-square displacement analysis on experimental trajectories **31**

15 List of measured gold nanoparticles **34**

1 Pearson correlation coefficient

We chose the Pearson correlation coefficient for several reasons. First, the Pearson correlation coefficient is normalized to the standard deviations of the signals, making it insensitive to the absolute amplitudes of the radial profile, therefore reducing the algorithm's sensitivity to size variations within the sample. This normalization ensures that our algorithm considers the overall geometry. Additionally, the Pearson correlation value ranges between -1 and 1, effectively distinguishing between correlated and anti-correlated signals. This is particularly beneficial in iSCAT, where axial modulations of the contrast have a periodicity of approximately $\frac{\lambda}{2}$. Depending on the application, other estimators can also be used with our method. In particular, multiple estimators can be used to further refine localization precision.

To calculate the Pearson correlation coefficient for two images of equal pixel dimensions, we consider each image as represented by two arrays, denoted as X and Y. These contain the pixel values of their respective images, which in this context present iSCAT contrasts. The Pearson correlation coefficient, r, is calculated using the following equation:

$$r = \frac{\sum_{i=1}^{n}(X_i - \overline{X})(Y_i - \overline{Y})}{\sqrt{\sum_{i=1}^{n}(X_i - \overline{X})^2}\sqrt{\sum_{i=1}^{n}(Y_i - \overline{Y})^2}}, \tag{S1}$$

where n is the total number of pixels in each image. X_i and Y_i are the pixel values of the ith pixel in images X and Y, respectively, and $\overline{X}$ and $\overline{Y}$ correspond to the mean pixel values of images X and Y, respectively. This equation normalizes the covariance of the two pixel arrays with the product of their standard deviations, ensuring that the correlation coefficient r lies in the range of -1 to 1. The case of $r = 1$ implies a perfect positive linear relationship between the pixel values of the two images.

2 Synthetic videos

We start by setting parameters that define the physical properties of the nanoparticles such as size and scattering cross section, as well as imaging conditions, such as frame rate and setup parameters, the medium in which the nanoparticle is suspended, and the diffusion coefficient (D). Next, we generate a 3D iPSF stack of the particle for different axial positions.

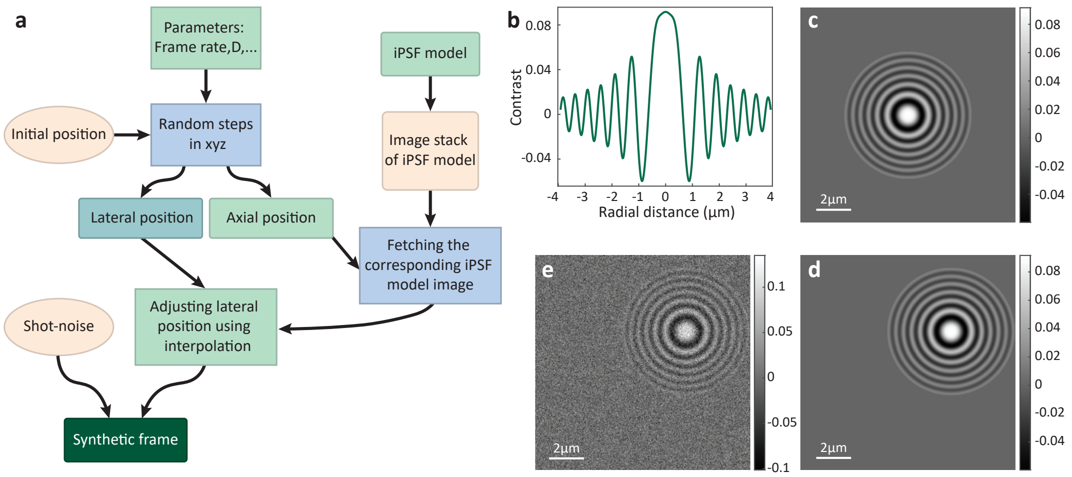

Figure S1: (a) A flow chart indicating the process of creating synthetic images. (b) An exemplary iPSF profile at the particle's axial position. (c) The image of the iPSF profile in (b). (d) The same as (c) but translated to the lateral coordinates of the particle using interpolation. (e) Final synthesized iSCAT image with added shot noise.

To simulate the Brownian motion of a nanoparticle, we employ Monte Carlo simulation. This begins with assigning an initial position in the 3D space (x, y, z) for the nanoparticle. By utilizing the predetermined frame rate, the simulation advances in discrete time steps. At each interval, the particle undergoes a random step, the magnitude and direction of which are determined by a random number with a normal distribution with a mean value of 0 and standard deviation of $\sqrt{2D\Delta t}$, where Δt is the time between two consecutive frames. The collection of these steps generates a trajectory that represents the Brownian motion of the particle in the medium.

For each position of the trajectory, the iPSF corresponding to the particle's current axial position is retrieved from the computed iPSF stack (FigureS1b) and its 2D image is

generated (Figure S1c). Next, the images from the iPSF stack are positioned at the lateral coordinates of the particle by a 2D interpolation. This method guarantees sub-pixel accuracy in the simulation, as illustrated in Figure S1d. To accurately replicate the conditions of our imaging apparatus, shot noise is incorporated into the synthetic data based on the full electron well capacity of our camera (Figure S1e).

3 Impact of design values of microscope objective on iPSF

In the iPSF model, mainly the refractive indices for immersion oil (n_o) and glass (n_g) are critical due to their influence on optical path differences and the resulting interference at the detector. In the main manuscript, we presented a method to calibrate the setup by measuring the iPSF as one varies the focal plane of the object while the GNP remains at the interface. The experimental results are then compared with the simulated models to estimate the setup parameters.

In Figure 1d of the main manuscript, we presented the correlation values between the experimental iPSF and the outcome of the model as a function of n_g and n_o. The maximum correlation value was chosen to model the iPSF of our setup. Yet, as shown in Figure 1d, a range of values exists along a stripe in the correlation matrix that gives similar correlation values close to 1. This stripe follows the values n_o and n_g, in which the accumulated phase along the propagation of the scattered light to the objective remains constant.

To investigate whether different choices of the refractive index pairs within that stripe would affect the resulting iPSF stack, we generated iPSF stacks for a range of axial positions of a 40 nm GNP using three distinct pairs of refractive indices on the aforementioned stripe. Figure S2 illustrates the iPSF stacks for three separate pairs of refractive indices along the diagonal with the highest correlation value (Figure S2a). Figure S2b, c, d displays the iPSF stacks for these three refractive indices pairs, specifically [$n_o = 1.5165$, $n_g = 1.5326$], [$n_o = 1.5217$, $n_g = 1.5285$], and [$n_o = 1.5286$, $n_g = 1.5233$], respectively. This sensitivity analysis indicates that while the refractive indices of immersion oil and glass are crucial for iPSF modeling, within certain refractive index ranges, the overall iPSF pattern remains remarkably consistent despite variations in individual values. The correlation between stacks was calculated to be over 99%.

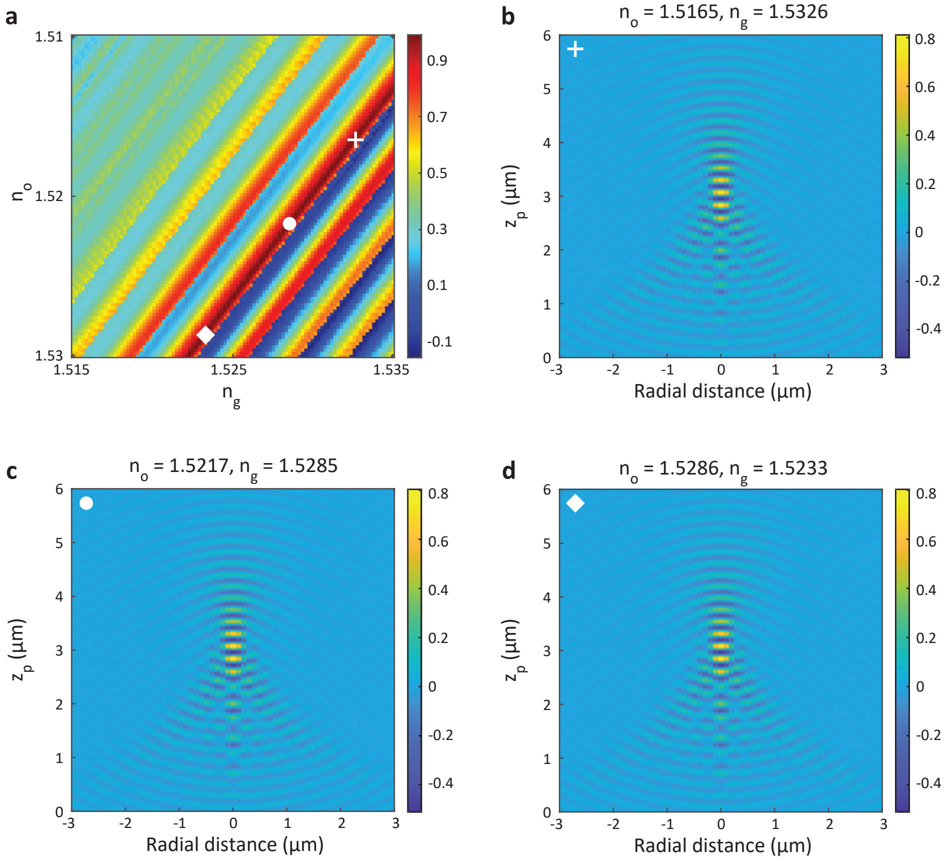

Figure S2: (a) Visualization of the correlation matrix between experimental iPSF and computational models across a range of refractive indices for immersion oil and glass (40 nm GNP at the water-glass interface and varying focal plane). The diagonal line indicates the series of index pairs where the sum $n_o + n_g$ remains constant, yielding the highest correlation values. (b-d) iPSF stack simulations for a 40 nm GNP at various axial positions using three representative pairs of refractive indices from the highlighted line in (a): $n_o = 1.5165$, $n_g = 1.5326$ in (b), $n_o = 1.5217$, $n_g = 1.5285$ in (c), and $n_o = 1.5286$, $n_g = 1.5233$ in (d). The consistently high correlation of over 99% demonstrates the insensitivity of the iPSF model to these particular refractive index variations, emphasizing the model's reliability for axial localization.

4 Impact of immersion oil refractive index on focal plane asymmetry in iPSF

In an aberration-free optical setup, the point spread function exhibits a symmetrical shape above and below the focus.[1] However, spherical aberrations are always present when the focal plane of an oil-immersion commercial microscope objective is set at a location away from the interface between the cover glass and water. In iSCAT, we benefit from this effect for breaking the axial asymmetry and, thus, determining the direction of particle motion.[2,3] This asymmetry can be enhanced by setting the refractive indices of the immersion oil and cover glass away from the designed values of the microscope objective.

To show the effects of spherical aberration on the correlation map and 3D tracking, we have modeled the iPSF stack of a 40 nm GNP for different refractive indices of the immersion oil ($n_i = [1.52, 1.51, 1.50]$). Figures S3a-c show the resulting iPSF stacks for different axial positions of the GNP, ranging from 0 to 8 µm. As n_i deviates from the designed value of the objective, the asymmetry of the iPSF becomes more pronounced. Using these iPSF stacks, we have synthesized the correlation maps of a diffusing particle (see Figures S3d-f) for the same axial trajectory (Figure S3g).

The iPSF stack in Figure S3a has the highest partial symmetry between two sides of the focal plane among the generated iPSF stacks. Therefore, its corresponding correlation map includes stripes around $2z_f - z_p$ with values just slightly smaller than the maxima in the stripes around the actual position of the particle (z_p). As spherical aberration becomes more pronounced in Figures S3b,c, the local maxima of the stripes near $2z_f - z_p$ decrease. Figure S3h shows line cuts of the correlation maps at frame number 1000. It is evident that the difference in the correlation values above and below the focal plane becomes larger with higher spherical aberration.

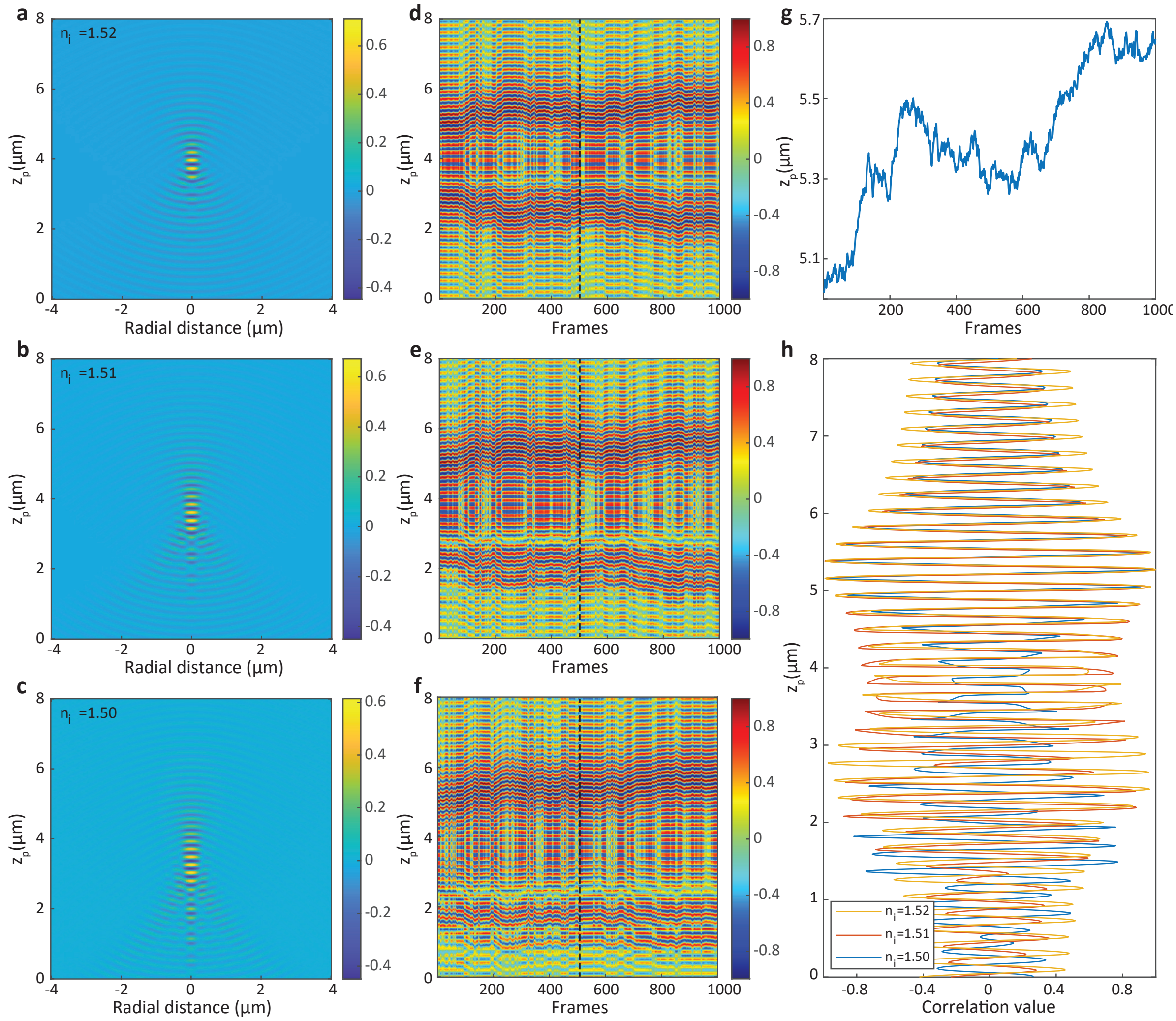

Figure S3: (a-c) iPSFs for 40 nm GNP in water with focal plane at 4 µm and designed immersion oil refractive index of 1.515. The immersion oil refractive indices are 1.52, 1.51, and 1.50 for (a-c), respectively. Color bars show the normalized iSCAT contrast. (d-f) Correlation maps of a diffusing particle with different immersion oil refractive indices corresponding to (a-c), respectively. Color bars present the normalized correlation values. (g) Axial position of the particle in the correlation maps of (d-f). (h) Correlation values of the frame number 500 in (d-f).

5 Effect of particle position on correlation map

The shape of the correlation map at a given frame is intricately linked to the axial position of a particle relative to the focal plane. As iPSF has partial symmetry on both sides of the focal plane, for a particle situated at z_p it is expected that the correlation with the iPSF model will exhibit local maxima near the values of z_p and $2z_f - z_p$. Spherical aberration as elucidated in our previous work[3] induces partial asymmetry in iPSF, allowing one to determine whether the particle is above or below the focal plane.

Figure S4a shows a correlation map of a diffusing particle when the particle is around 1.5 µm above the focal plane. Figure S4b shows the axial position of the particle corresponding to Figure S4a. As we observe in Figure S4c, there are two dominant peaks of the correlation values on both sides of the focal plane. Due to the spherical aberration of the setup, these values are different by around 10%. In the case that the particle diffuses near the focal plane (see Figures S4d,e), due to the limited spatial features of iPSF, the difference between the first and the second peaks becomes smaller (Figure S4f). Here, if one aimed to find the axial position of the particle using the information in only one frame, the result would be erroneous as the maxima of the correlation values can jump by the distance of $\frac{\lambda}{2}$ from frame to frame due to various factors, including shot noise. Yet, our algorithm performs robustly because it uses the sum of the correlation values of multiple frames in a branch.

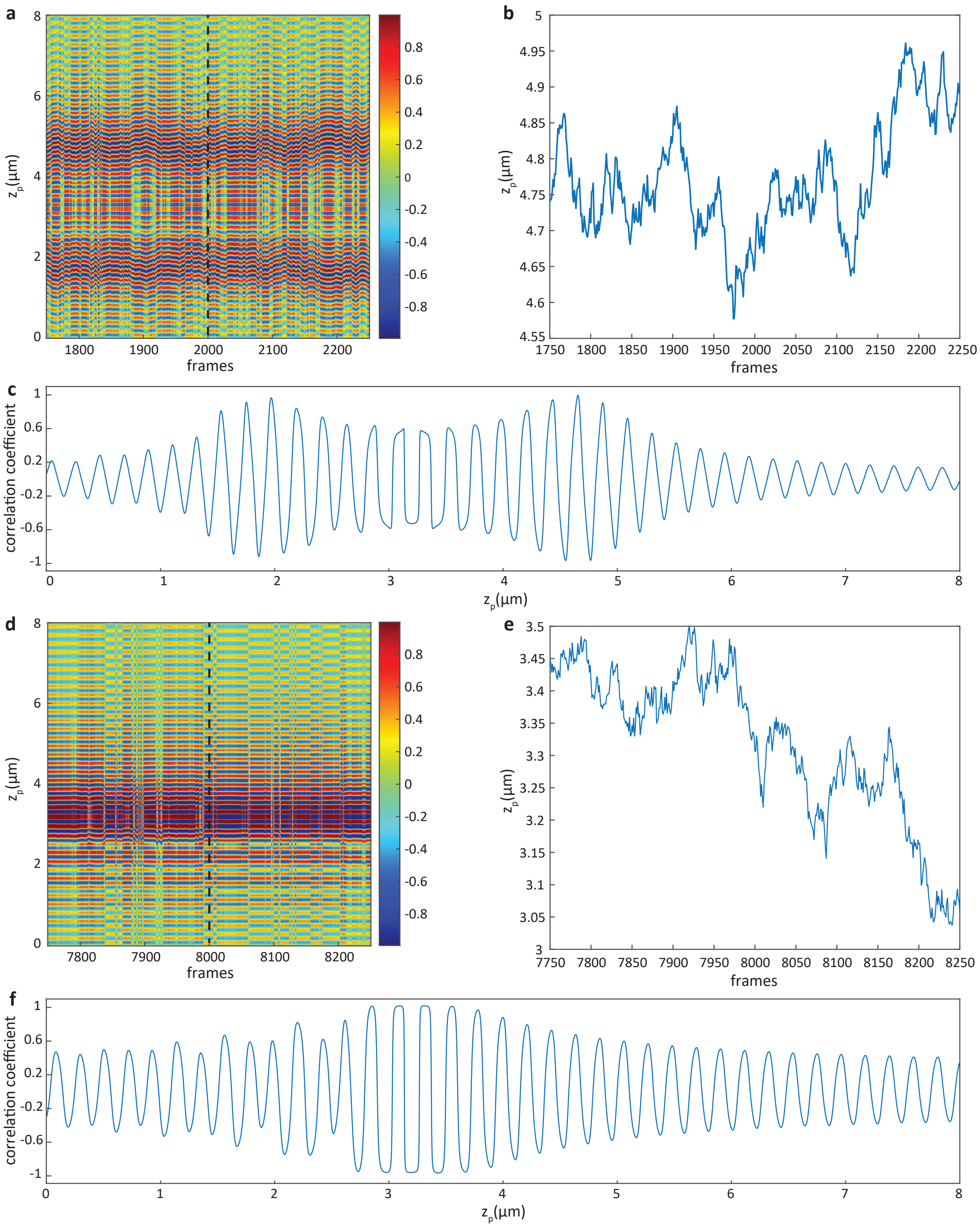

Figure S4: (a) Correlation map of a diffusing particle when the particle is away from the focus, for $z_f = 3.2\,\mu\text{m}$. (b) Particle's axial position corresponding to (a). (c) Correlation values for frame number 2000 in (a). (d) Correlation map of a diffusing particle when the particle is near the focus, for $z_f = 3.2\,\mu\text{m}$. (e) Particle's axial position corresponding to (d). (f) Correlation values for frame number 8000 in (d). In (a,c) the color bars represent the normalized correlation values.

6 Boundary tracing of correlation map

The values of a correlation map are distributed symmetrically around zero. This is due to the axial modulation of the iPSF with a periodicity of $\sim \frac{\lambda}{2}$. Hence, an iPSF with an axial distance of $\frac{\lambda}{4}$ from the true position of the particle exhibits a correlation value near -1. Figure S5a shows the correlation map, illustrating this axial modulation. To examine the distribution of these values, Figure S5b presents the histogram of the correlation map, confirming the symmetry of the values around zero. To further analyze the correlation map, we first binarize the values by setting the values above zero to 1 and turning the negative values to zero. Figure S5c shows an example of a binarized correlation map.

We use the Moore-neighbor boundary tracing algorithm[4] for identifying and outlining the boundaries of regions in our binarized correlation map. The algorithm begins by pinpointing an initial boundary pixel and systematically traverses the region to establish its boundaries. Incorporating Jacob's stopping criterion,[5] the algorithm halts when it returns to the pixel that initiated the boundary detection, thereby defining a region along with its boundaries. This iterative process enables the algorithm to detect all interconnected regions within a given image. In MATLAB, the function *bwboundaries* is employed for such boundary tracing tasks. Additionally, the connectivity of pixels can be adjusted. In our 3D tracking algorithm, we utilized an 8-pixel connectivity configuration. Figure S5d depicts the boundary tracing algorithm on the binarized correlation map shown in Figure S5d.

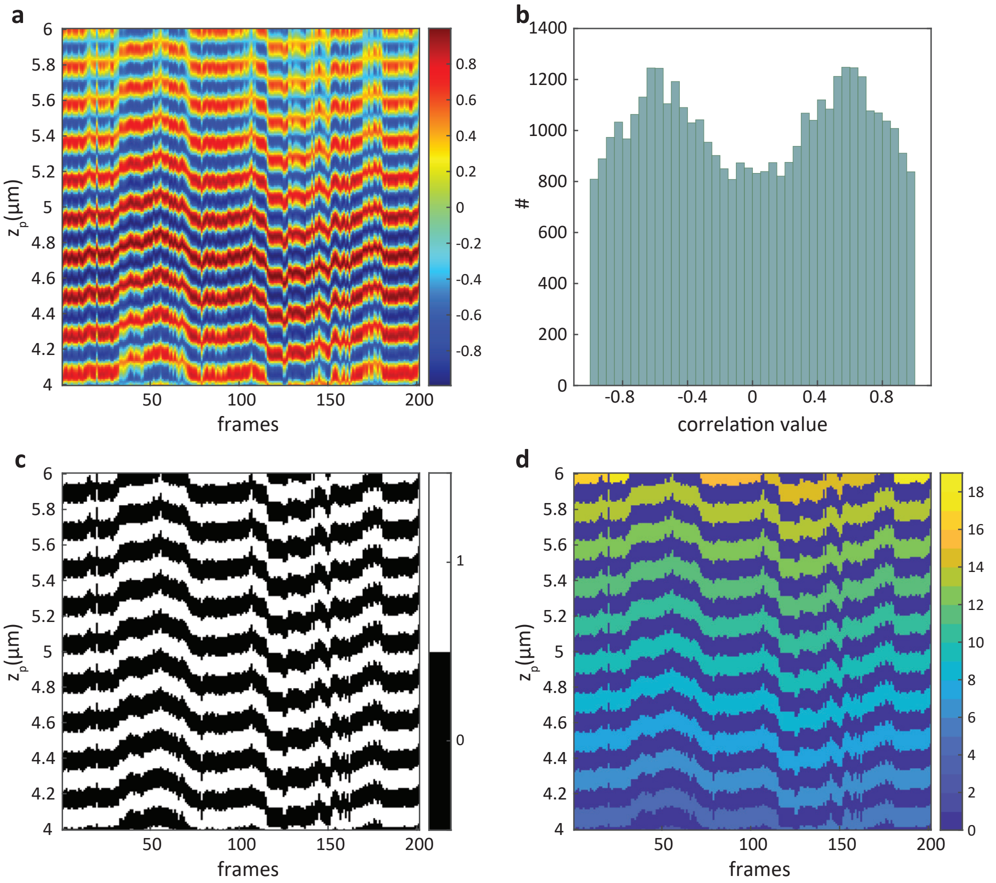

Figure S5: (a) An example of a correlation map of a diffusing particle. Color bar represents the normalized correlation values. (b) Histogram of the values of the correlation map in (a). (c) Binarized version of (a). (d) Result of the boundary tracing of (c), detecting regions of the correlation map. Color bar shows the integer indices assigned to each region.

7 Graph representation of the correlation map

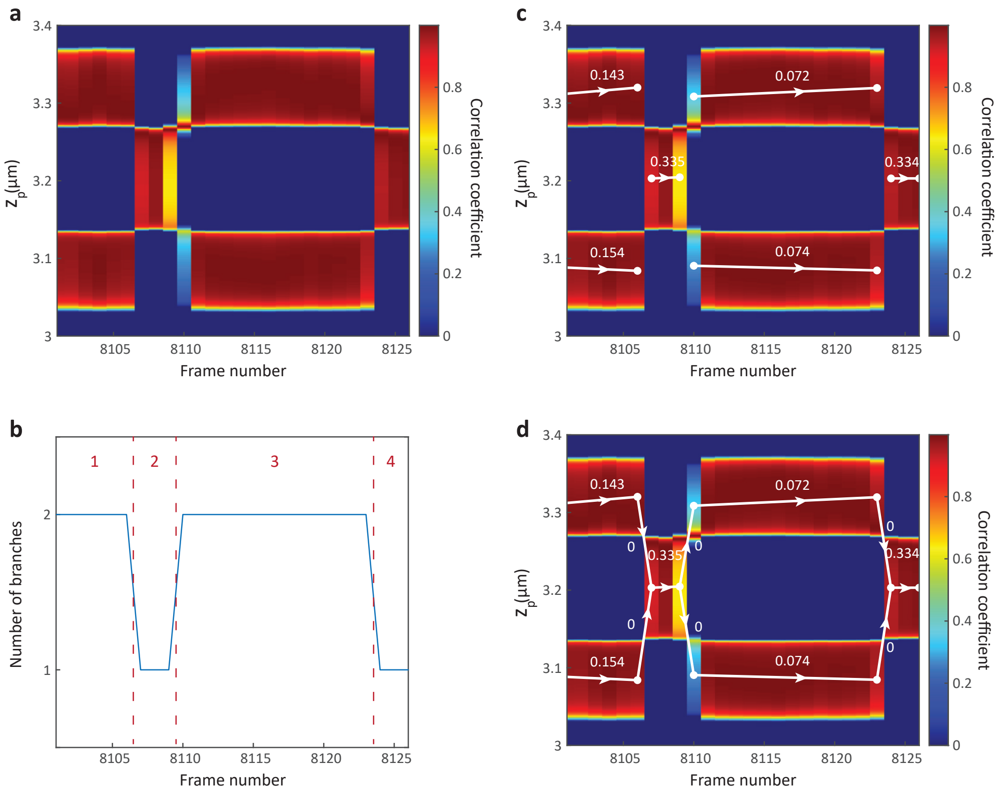

Figure S6: (a) Exemplary correlation map of the selected region (from Figure 3 of the main text). (b) Number of branches over frames, with branching points marked by dashed lines. (c) Graph construction within the isolated regions with nodes at branching points and edges representing distances. (d) Connected graph.

To accurately determine the axial position of a particle crossing the focal plane, we utilize a graph representation of the correlation map. Here, we detail the process of constructing and utilizing this graph representation for precise axial tracking. For this process, we start with the selected region of the correlation map, i.e. Figure 3b of the manuscript, and perform the processes mentioned below to create the graph representation from the selected region of the correlation map. Branching within the selected region complicates the determination of the maximum correlation value for axial localization. An instance of branching within the selected region is depicted in Figure S6a.

Identifying branching points. Branching points, where the number of branches changes, are identified by monitoring the change in the number of branches from frame i to frame $i+1$ (see Figure S6b). Then the correlation map is divided into isolated regions where the number of branches remains constant (indicated with integer numbers in Figure S6b).

Graph construction within regions. Each branching point represents a node in the graph. The distance between two connecting nodes represents the edge of the graph. We start with constructing the graph within each isolated region. For example, in region 1 of Fig S6b, we have two branches. Therefore, we have two edges. Each edge has a distance b_m, calculated as in Eq.5 of the manuscript. Figure S6c shows the nodes and edges of all the isolated regions. Then, the nodes of the consecutive isolated regions are connected with a distance of 0 (see Figure S6d), creating a connected graph throughout the correlation map.

Distances b_m are inversely related to correlation values, meaning the path with the minimum distance includes the highest correlation values (see Figure S6d). The edges' direction reflects the chronological order of frames.

Retrieving the coarse axial localizations by finding the shortest path in the graph. Now that the selected region of the correlation map is represented by a directional graph, we use Dijkstra's algorithm[6] to find the shortest path between any two nodes in the graph. The initial node is set to the frame where the nanoparticle first appears in the video, and the final node is set to the frame where the nanoparticle is last observed. The final single-branch correlation map is reconstructed from this shortest path.

8 Extended tracking capabilities across the focal plane

Our algorithm is capable of tracking nanoparticles as they move through and across the focal plane. Figure S7 exemplifies this capability with a 40 nm GNP as it traverses the focal plane set at 15 µm. As shown in Figure S7a, the iPSF stack is asymmetric about the focal plane due to the spherical aberration. Figure S7b depicts a 40 nm GNP trajectory crossing from below to above the focal plane retrieved by our method. Figure S7c features the correlation map associated with this trajectory, whereby Figure S7d depicts cross-section of the correlation map 3 µm below and above the focal plane, with the maximum correlation values marked by red arrows. In Figure S7e, the measured and modeled iPSFs for the axial positions corresponding to the maximum correlation values in (d) are compared.

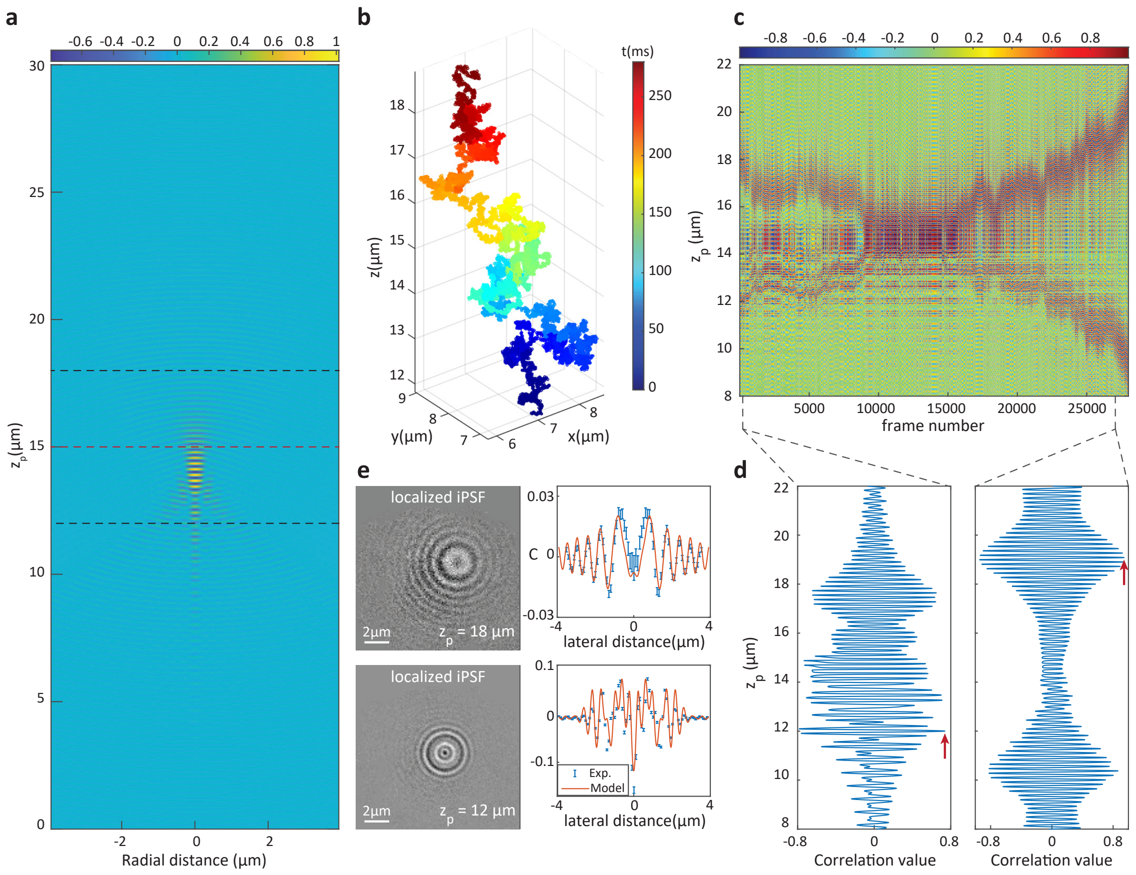

Figure S7: (a) iPSF model 40 nm GNP ($z_f = 15\,\mu$m) (b) A trajectory crossing the focus. (c) The correlation map of (b). (d) Cross sections of the correlation map, where the particle is below (left) and above (right) the focal plane. The maximum correlation values are indicated with red arrows. (e) The corresponding iSCAT frames, along with their overlays using the iPSF model's radial profiles for particles with the highest correlation values in (d). The error bars in the radial profiles represent the shot noise level at each radial distance.

9 Long axial range 3D tracking on synthetic videos

To demonstrate the capability of our algorithm in tracking nanoparticles over a large axial range, particularly those distant from the focal plane, we used a Monte Carlo simulation to model the diffusion of 30 nm and 60 nm GNPs in water. The simulation outputs (the 3D position of the particles) were then used to generate synthetic videos that replicate our measurement conditions, including shot noise. These videos, captured at a high frame rate of 100 kHz, were created with a shot noise level σ_n set to 0.013, which is twice the expected noise at full electron-well capacity in our experimental setup.

Figures S8 and S9 present the results for 60 nm and 30 nm GNPs with the focal point at z_f=15 µm for both cases, respectively. The histograms in Figures S8 and S9 compare the localization errors along the x, y, and z axes, revealing that the axial localization error is notably smaller than the lateral errors. This finding is consistent with the results shown in Figure 4d of the main manuscript.

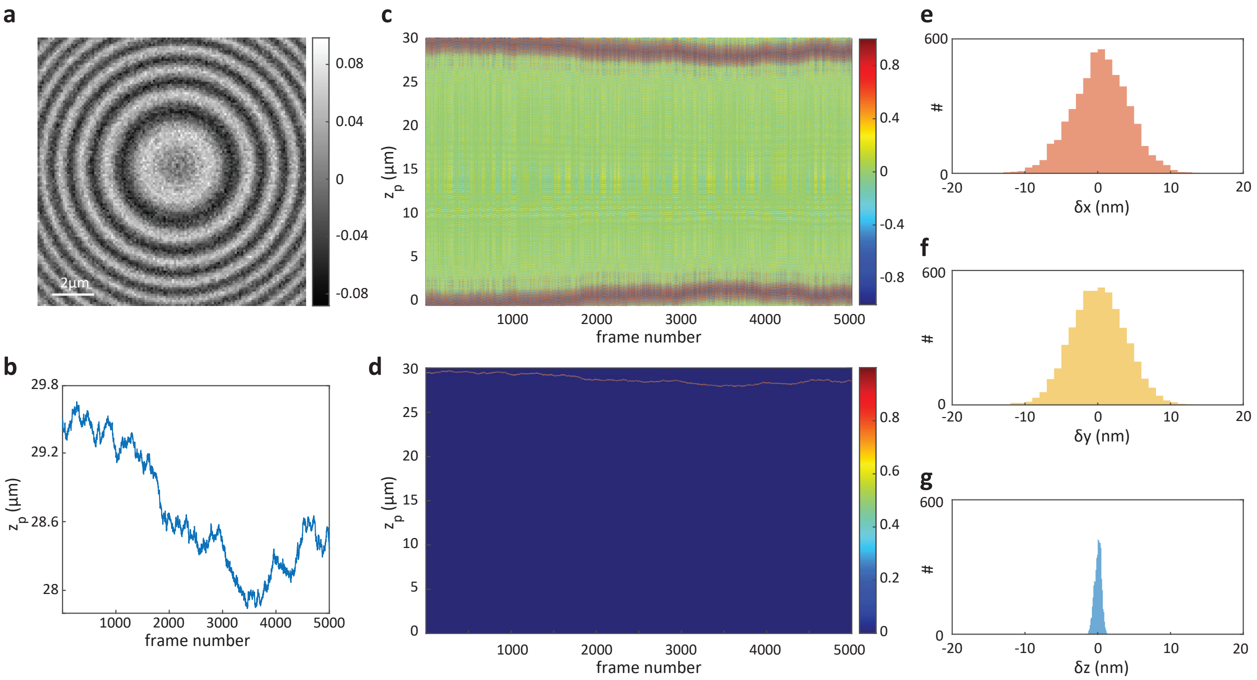

Figure S8: (a) A frame of the synthesized video of a 60 nm GNP far from the glass interface (≈ 29.5 µm) with focus at z_f = 15 µm. (b) Ground truth of the particle's axial position in the trajectory. (c) The correlation map of the trajectory. (d) The selected region of the correlation map is in (c). (e-g) Localization errors along each of the three axes.

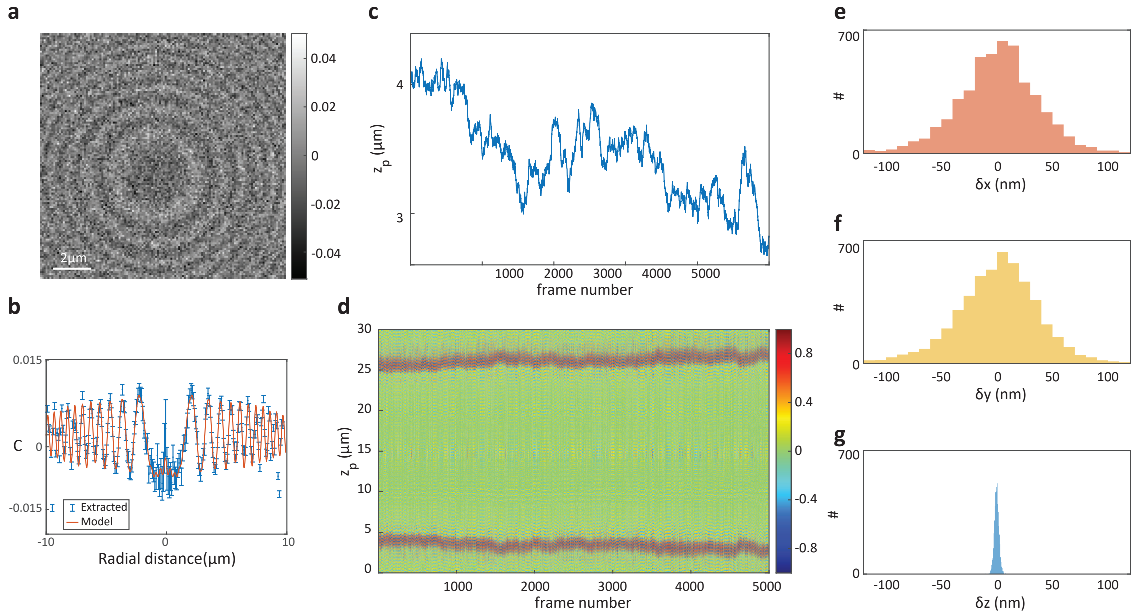

Figure S9: (a) A frame from the synthesized video of a 30 nm GNP near the glass interface (approximately 4 µm) with the focus at $z_f = 15$ µm. (b) The extracted iPSF profile overlaid with the ground truth radial profile from (a), showing the radial profile spanning the entire radial extent of 10 µm. The error bars in the radial profiles represent the shot noise level at each radial distance. (c) Ground truth of the particle's axial position in the trajectory. (d) The correlation map of the trajectory, resulting from the correlation of the full-extent radial profiles with those of the iPSF model. (e-g) Localization errors along the three axes, with axial tracking performed on extracted radial profiles with 10 µm radial extent.

10 Long axial range 3D tracking using truncated iPSF profiles

In Figures S8 and S9, axial tracking was achieved by using the full extent of the radial profile of the iPSFs (10 µm). However, it is also possible to track the axial position of the particle using a truncated radial profile. To understand how the chosen extent of the radial profile affects the axial tracking of the nanoparticle, we also explored tracking using truncated radial profiles of the iPSFs. Figure S10 presents the results of tracking the same nanoparticle shown in Figure S9, but with a radial extent limited to 4 µm. A comparison between Figures S9g and S10e reveals no significant changes in the axial localization errors. We attribute this to the fact that the signal-to-noise ratio decreases at greater distances from the center, due to

the weaker signal in the outer rings.

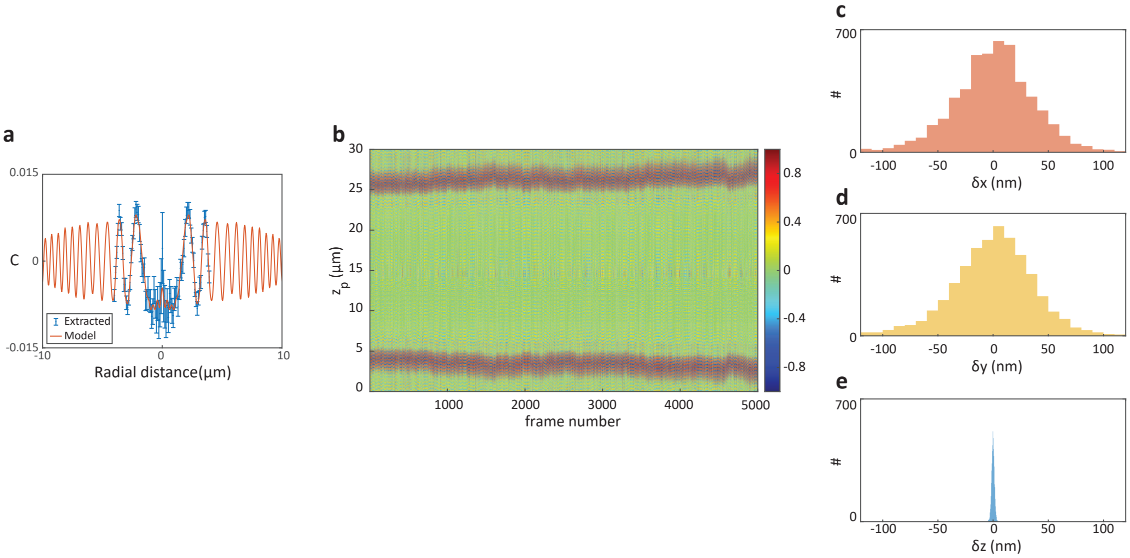

Figure S10: (a) The extracted iPSF profile, with a radial extent of 4 µm, overlaid with the ground truth radial profile, which has a radial extent of 10 µm. Error bar represents the shot noise level at each radial distance. (b) The correlation map of the trajectory, generated by correlating the extracted radial profiles with a 4 µm extent and those of the iPSF model. (c-e) Localization errors along the three axes, with axial tracking performed on extracted radial profiles with 4 µm radial extent.

11 Determining the axial range experimentally

To determine the axial tracking range of GNPs experimentally, we systematically recorded the iPSFs of GNPs of 80 nm, 60 nm, 40 nm, 30 nm and 10 nm suspended in water. We collected data for more than 100 particles per size category. For the GNPs of diameter 80 nm, 60 nm, 40 nm, and 30 nm, the focal plane was positioned 15 µm above the glass-water interface to facilitate the measurement of the axial range. For the 10 nm GNPs, the focal plane was positioned 1 µm above the interface. The 3D trajectories of these GNPs were then retrieved using our localization algorithm, as explained in the manuscript.

Figure S11 presents the experimental data for GNPs of varying sizes. In panels (i) and (ii), we display exemplary trajectories of GNPs located at the furthest measured distances from the focal plane, (i) showing the trajectory above and (ii) below the focal plane, respectively. Panels (iii) and (iv) illustrate the corresponding iPSF images for the particles at these extreme axial positions. Data acquisition for the 80 nm GNPs was performed at 70 kHz, whereas 60 nm, 40 nm, and 30 nm GNPs were tracked at 100 kHz. For the smallest GNPs (10 nm), the acquisition rate was 200 kHz to accommodate the higher diffusion. We note that the experimentally determined axial range (e.g., 30 µm range observed for 80 nm GNPs) is not the fundamental limit in our imaging system. Indeed, a closer look at the contrast level of the iPSF at the most distant axial location reveals that the signal-to-noise level is still sufficient to track the particle at larger distances. However, the current FOV restricts our capacity to entirely capture these highly defocused iPSF images. This constraint is not fundamental to the method but rather a limitation of the present setup.

The ability to track particles over such long axial ranges relies on the reliable modeling of the spherical aberrations caused by the particle being in water. To further elaborate on the accuracy of the iPSF model, we have depicted, in Figure S12, the correlation map and the radial profiles of the 80 nm GNP in Figure S11a(i), overlaid with the corresponding radial profiles of the iPSF model. In Figure S12, we observe a strong agreement between

the experimental and modeled iPSFs, which confirms the accurate modeling of the spherical aberration.

Table S1: Maximum axial ranges for GNPs of different sizes.

Size	Maximum range below focal plane (µm)	Maximum range above focal plane (µm)
80 nm	14.7 µm	15 µm
60 nm	14.8 µm	14.9 µm
40 nm	10.2 µm	12.2 µm
30 nm	5.1 µm	10.1 µm
10 nm	1 µm	2.7 µm

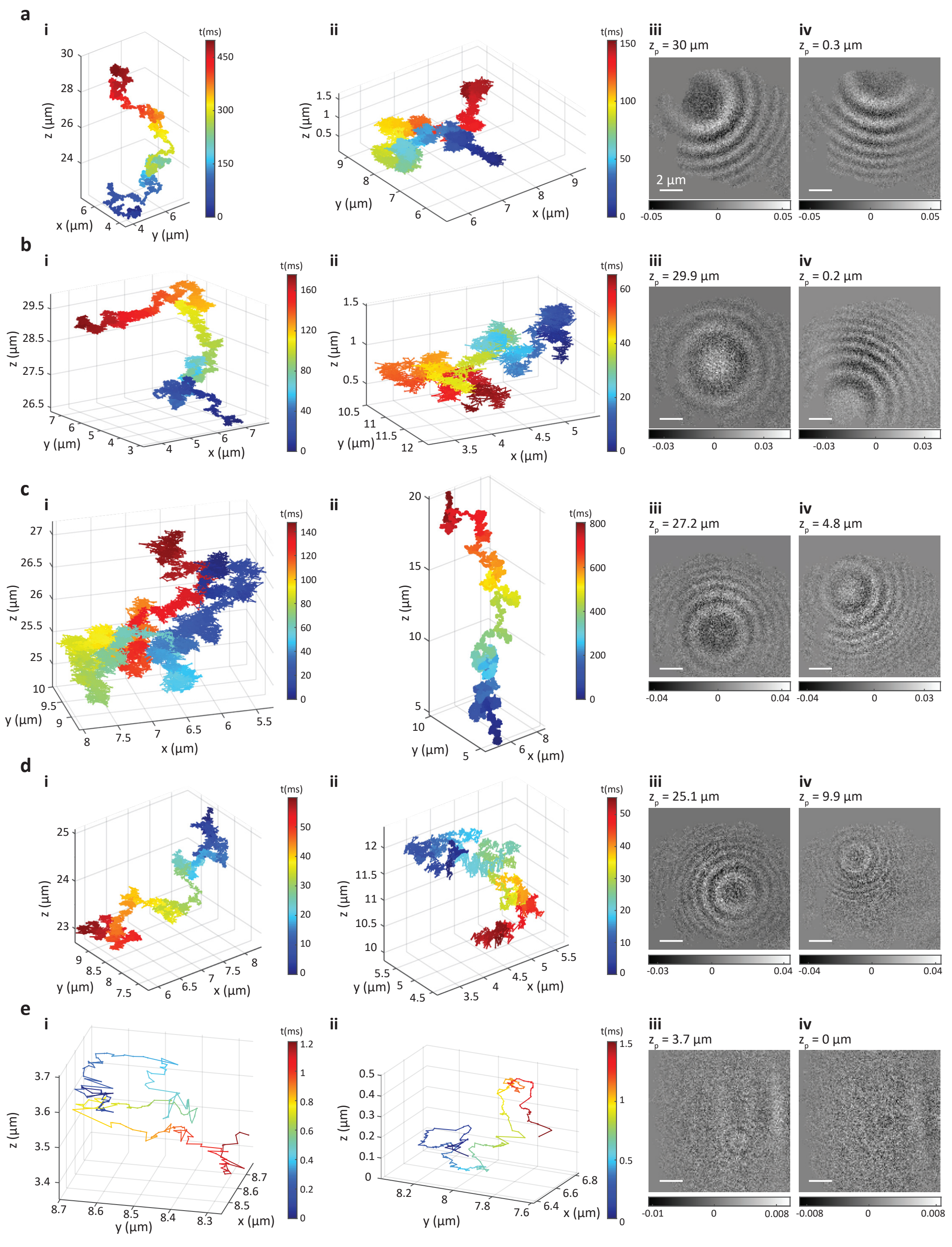

Figure S11: Long axial range determination of GNPs. Panels (a-e) display results for GNPs with diameters of 80 nm, 60 nm, 40 nm, 30 nm and 10 nm respectively. Column (i) illustrates exemplary 3D trajectories of measured GNPs at the furthest distance above the focal plane, while column (ii) depicts 3D trajectories at the furthest measured distance below the focal plane. Columns (iii) and (iv) present the corresponding iPSF for these extremes in axial position, with z_f above each, indicating the particles' axial location relative to the glass-water interface. The images shown in (e)iii and (e)iv are averaged over 15 consecutive frames.

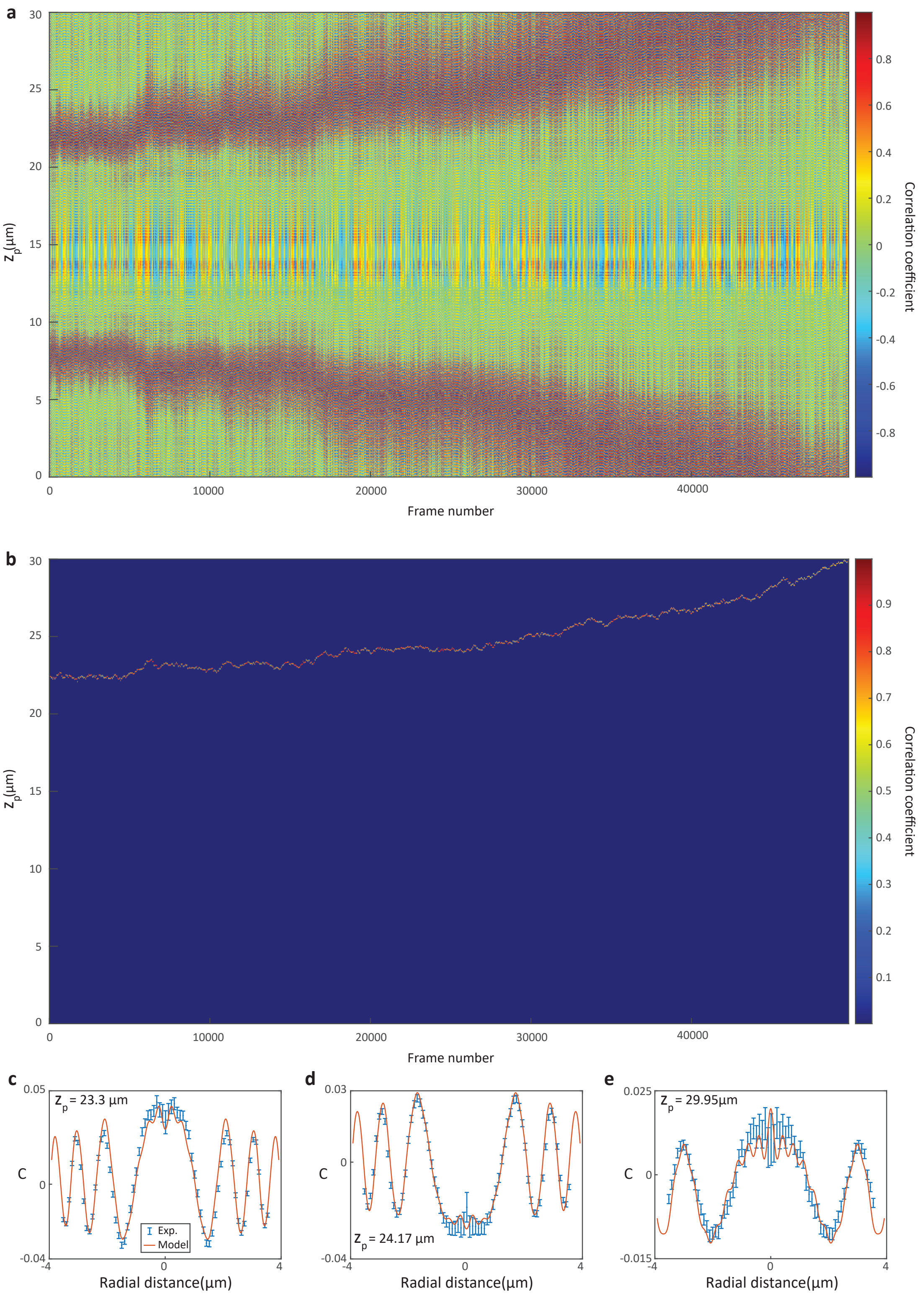

Figure S12: (a) Correlation map of the trajectory in Figure S11a (i). (b) Selected region of the correlation map in (a). (c-e) The overlaid experimental and modeled iPSFs. The error bars in the radial profiles represent the shot noise level at each radial distance. The corresponding iPSF models have correlation values of 0.98, 0.99, and 0.94, respectively.

12 Tracking GNPs with signal near noise level

Here, we present a detailed analysis of the tracking of diffusing 10 nm GNPs in water where their iSCAT signal is comparable to the shot noise level of a single frame in our measurements. As depicted in Figure S13a, the shot noise level prevents us from detecting the signal from the particle. To improve the SNR, we calculate the average of 15 consecutive frames, creating a moving average throughout the entire video. This improves the SNR by about $\sqrt{15}$. Figure S13b showcases an example of the improved signal from a 10 nm GNP.

The corresponding extracted radial profiles and the correlation map of the particle, as shown in Figure S11e(iii), are depicted in Figures S13c and S13d, respectively. The correlation coefficients in Figure S13d provide a quantitative measure of the tracking reliability, with high coefficients indicating strong agreement between the experimental data and the modeled iPSFs. It is important to note that when studying the mean squared displacement of such a trajectory, the effect of averaging should be taken into account to ensure an accurate interpretation of the particle dynamics.

To understand the effect of applying a moving average on the measurement of particle diffusion, we examine how the step size distribution changes with different time delays in Brownian motion. The mean square displacement (MSD) scales linearly with the time delay Δt. When calculating step sizes over different time delays $\Delta t = m\delta t$, where m is the number of frames and δt is the time interval between consecutive frames, the step size distribution broadens with increasing Δt. This broadening reflects the diffusive nature of the particle's motion, as larger time intervals allow for greater displacements.

Now, consider applying a moving average over N frames to the particle's trajectory. The averaged position at any given time t, denoted $x_{\text{avg}}(t)$, is calculated as:

$$x_{\text{avg}}(t) = \frac{1}{N} \sum_{i=0}^{N-1} x(t - i\delta t),$$

where $x(t)$ is the original position of the particle without the moving average. The moving

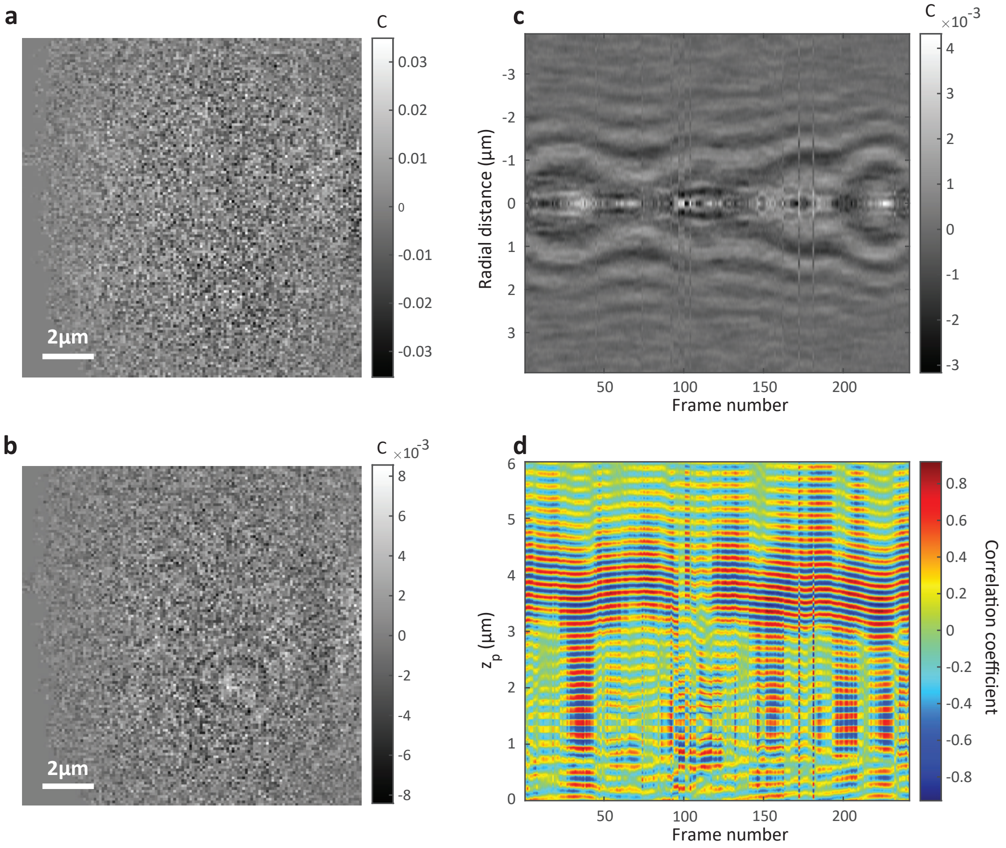

Figure S13: (a) A single frame image of the 10 nm GNP from the trajectory shown in Figure S11e(i). (b) The same frame after applying a 15-frame temporal moving average. (c) The extracted cross-section of the particle in Figure S11e(i). (d) The correlation map of the trajectory in Figure S11e(i)

.

average smooths the trajectory by averaging out fluctuations over the window of N frames. This smoothing effect reduces the variance of the step sizes calculated over short time delays (when $m \leq N$) resulting in a narrower distribution.

To demonstrate these effects, we synthesized a video of a particle with a diffusion coefficient similar to that of a 10 nm GNP ($D = 44.1\,\mu\text{m}^2/\text{s}$) in water, recorded at a frame rate of 200 kHz. In this video, the shot noise was not included in the simulation. Therefore, we were able to track the 10 nm GNP both with and without the moving average. In Figures S14 a-c, we compare the step size distributions for $m = 1$ (consecutive frames) in both conditions (with and without a $N = 15$ moving average). We observe that the SD of the step size distribution (σ) decreases when the moving average is applied, resulting in a narrower

distribution. This reduction is around $\frac{1}{\sqrt{N}} = \frac{1}{\sqrt{15}}$. Because the particle's position in all the axes would be the average of the frames included in the moving average. As shown in Figures S14 d-f, this narrowing effect is not present when comparing step sizes over $m = 30$ intervals, where the influence of the moving average becomes negligible. In Figures S14 g-j, we compare the MSD plots for the video with and without the moving average. After a time interval corresponding to $N = 15$ frames (i.e., greater than $0.075\,\mathrm{ms}$), the MSD curves for both conditions become parallel. This indicates that, using the data points of the MSD curves where $m \leq N$, would lead to inaccurate estimation of the diffusion, but using the data point where $m > N$ would yield the correct results.

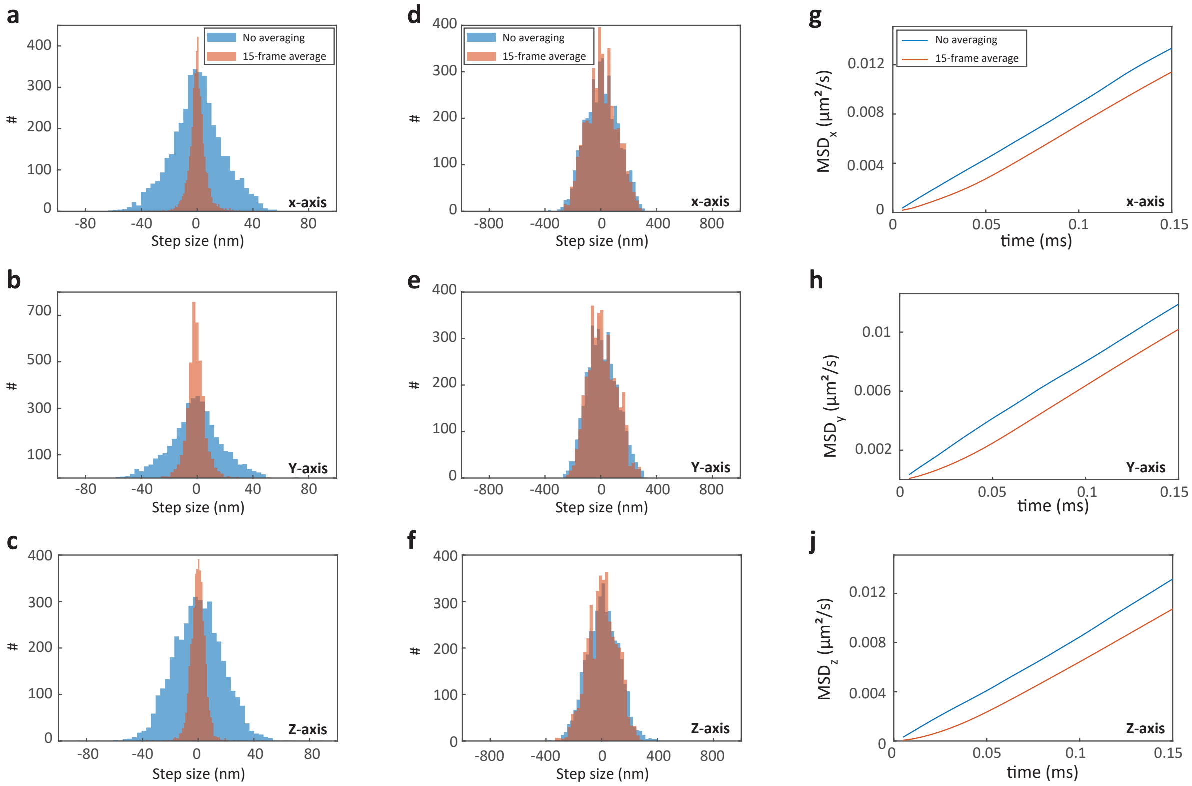

Figure S14: (a-c) $m = 1$ step size distributions of the 10 nm GNP along all three axes. The standard deviations of the step size distributions are $\sigma = 18.51$ nm and $\sigma_{avg} = 7.44$ nm for the x-axis, $\sigma = 18.38$ nm and $\sigma_{avg} = 7.69$ nm for the y-axis, and $\sigma = 17.60$ nm and $\sigma_{avg} = 6.99$ nm for the z-axis. (d-f) Step size distributions of the 10 nm GNP over $m = 30$ intervals along all axes. The SD of the step size distributions are $\sigma = 115.4$ nm and $\sigma_{avg} = 106.7$ nm for the x-axis, $\sigma = 108.1$ nm and $\sigma_{avg} = 99.9$ nm for the y-axis, and $\sigma = 114.3$ nm and $\sigma_{avg} = 103.6$ nm for the z-axis. (g-j) MSD of the tracked particle with and without the moving average along all three axes.

.

13 Tracking multiple nanoparticles in the iSCAT field of view

In the preceding section, we presented trajectories spanning a significant axial range of over 30 µm. Illustrated in Figure S11, the iSCAT signals from out-of-focus particles can occupy the entire FOV. Despite the wide lateral spread of individual iPSF profiles, our algorithm can effectively track multiple particles with minimal impact on performance. We confirm this by generating synthetic iSCAT images of two defocused particles at different axial distances

from the glass with varying lateral separations, as shown in Figure S15.

Figure S15a-e displays two particles with decreasing lateral distances, ranging from 9 µm to 0.52 µm. The particles positioned on the left and right sides of the FOV are at 7 µm and 23.5 µm above the glass, respectively, while the focal plane is at 15 µm. For lateral particle localization, we utilize the RVT algorithm, which leverages the circular symmetry of the iPSF. The extracted radial profiles, as depicted in Figure S15, are compared to the ground truth profiles (depicted for the left-side particle). Remarkably, even at distances smaller than 1 µm, as we average the radial profile across the iPSF rings, the extracted radial profile closely resembles the ground truth.

To assess the algorithm's localization capability, we calculate correlation values for each extracted radial profile using a stack of iPSF models containing radial profiles of particles that span from 0 µm to 30 µm axially. As indicated in Figure S15 a-d iv, the maximum correlation value corresponds to the true axial position of the particle. Conversely, when the lateral distance falls below 0.6 µm, the algorithm detects an axial location on the opposite side of the focal plane. These results show that even with a large lateral extent of the iPSF profiles, the required lateral separation remains below 0.6 µm.

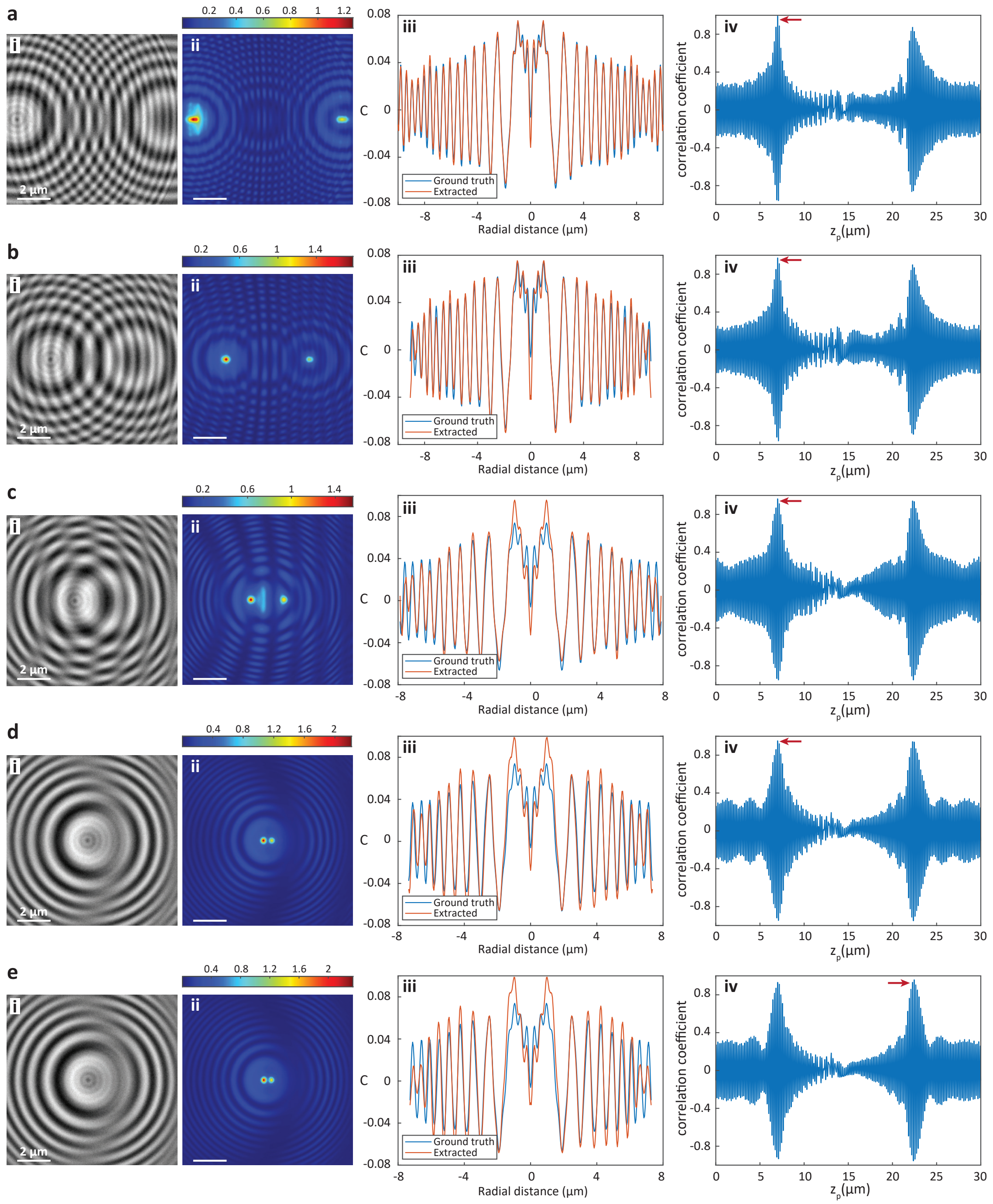

Figure S15: iSCAT imaging and radial profile extraction for two particles with decreasing lateral distances: 9 µm in (a), 5 µm in (b), 2.04 µm in (c), 0.6 µm in (d), and 0.52 µm in (e). Panel (i) displays the iSCAT signals of two particles defocused at axial distances of 7 µm (left particle) and 23.5 µm (right particle) from the glass, with the focal plane set at 15 µm. Panel (ii) depicts the output of the RVT algorithm for the lateral localization. Panel (iii) shows the extracted radial profiles compared with the ground truth profiles for the left-side particle. Panel (iv) presents the correlation values of the extracted radial profile with the iPSF model. Red arrows indicate the maximum value of the correlation.

14 Mean-square displacement analysis on experimental trajectories

To understand the diffusive behavior of the GNPs and determine the experimental localization precision of our algorithm, we conducted a detailed analysis of their mean square displacements (MSDs) in three dimensions. The analysis of the MSD versus time allows for the extraction of key parameters: the diffusion constant (D) and the dynamic localization error (σ_{loc}). For each trajectory, we calculated MSD for different time steps from 1 frame (14.3 µs for 80 nm GNP and 10 µs for 40 nm GNP) to 1 ms for each directional component. The thin lines in Figure S16 (a-c) and (e-g) represent the MSDs in the x, y, and z directions for 80 nm and 40 nm GNPs, respectively. The thick lines represent the averaged MSDs over the ensemble trajectories. In Figure S16 (d) and (h) the MSD plots are 80 nm and 40 nm. The MSD plot is fitted to a linear function, $\mathrm{MSD}(t) = a + bt$ as explained in Ref.[7] The diffusion constant is derived as $D = b/2$ for the 1D projection. The offset (a) is associated with the dynamic error in the localization with the following formula:

$$\sigma_{\mathrm{loc}} = \frac{1}{2}\sqrt{a + \frac{bt_{\mathrm{exp}}}{3}},$$

where t_{exp} is the camera exposure time. Figure S16 depicts the results. Figure S16d, g illustrates the linear fit to the average MSD curves for 80 nm and 40 nm GNPs, respectively. The z-direction exhibits a notably lower y-intercept, indicative of a more precise localization in the axial dimension compared to the x and y lateral directions. For 80 nm GNPs, the x, y, and z directional localization errors were found to be 7.6 nm, 7.2 nm, and 4.4 nm respectively. For 40 nm GNPs, the corresponding errors were 9.5 nm, 8.8 nm, and 4.6 nm. The lateral localization error is consistent with the previously reported analysis.[8] For the other GNPs of different sizes, we also observe that the experimental axial localization error is substantially less than the lateral axes. The MSD curves of the GNPs with diameters of

60 nm, 30 nm, and 10 nm along the three axes are depicted in Figure S17.

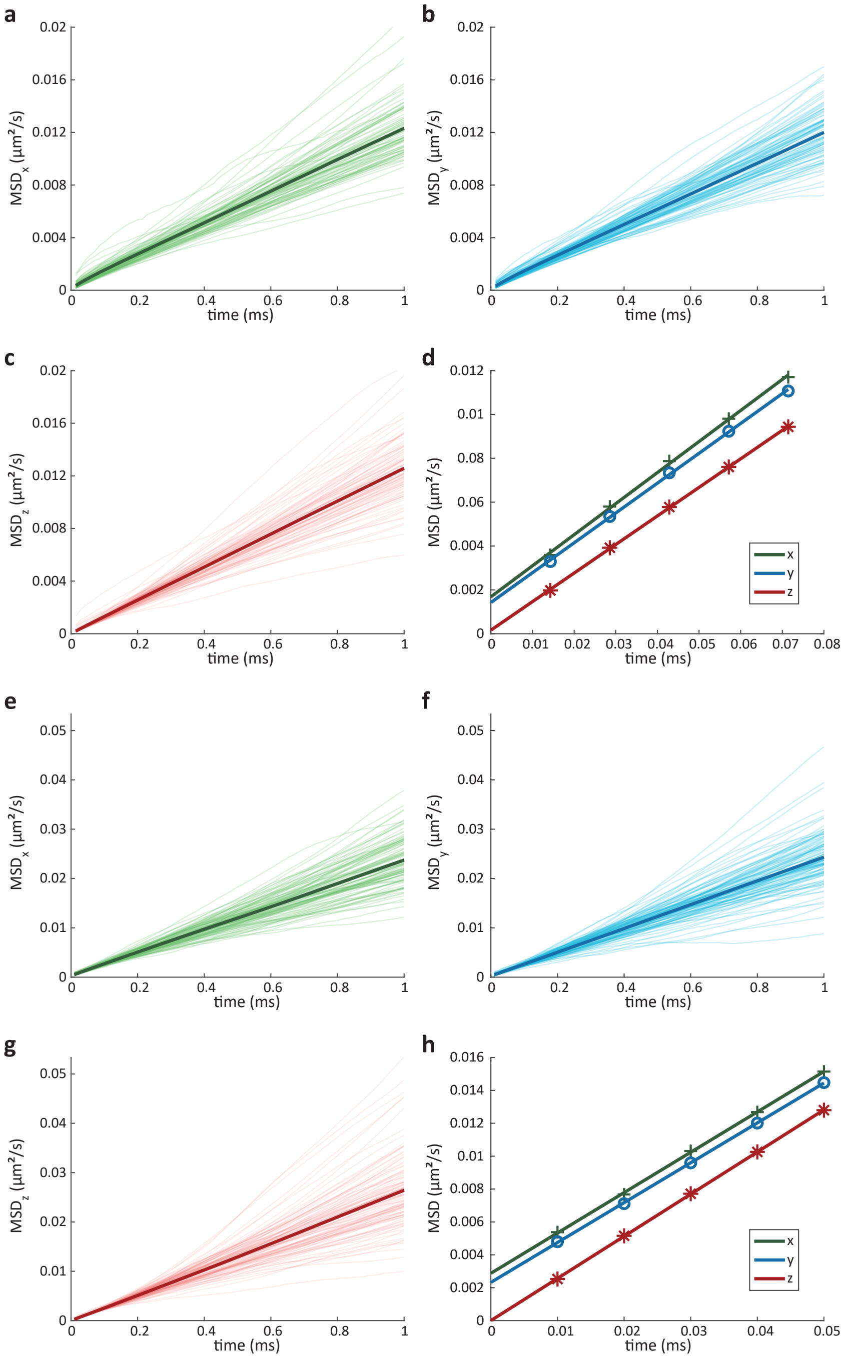

Figure S16: Mean square displacement analysis for GNPs. (a-c) MSD curves along the x (a), y (b), and z (c) directions for 80 nm GNPs, with each thin line representing MSD for an individual trajectory, and the thick line denoting the average over the MSD curves. (d) The linear fit to the averaged MSDs from (a-c). The lower y-intersection for the z-component confirms the smaller localization error in comparison to the lateral direction. (e-h) The same analysis for trajectories of 40 nm GNPs in water in the x (e), y (f), and z (g) directions, respectively. The linear fit to the average MSD curves is displayed in (h).

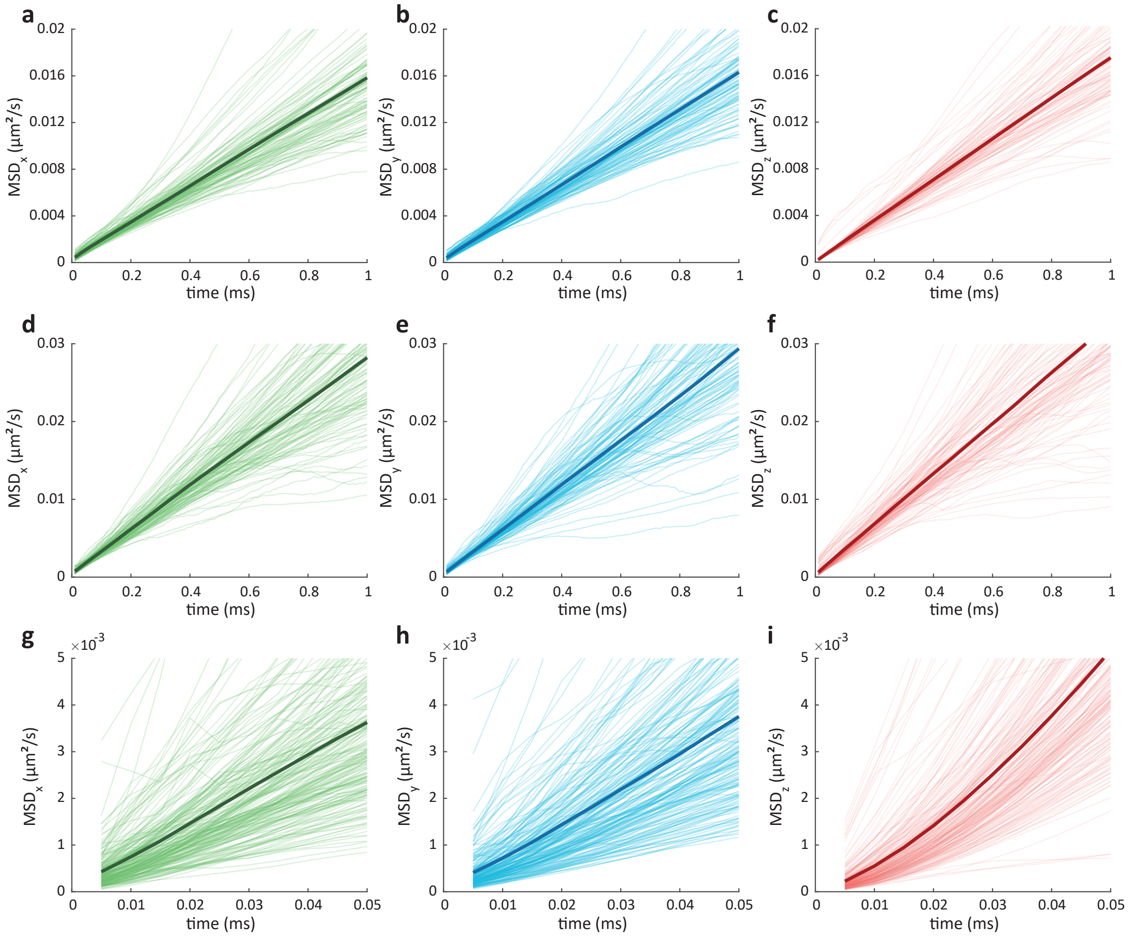

Figure S17: Mean square displacement curves for GNPs of different diameters: (a-c) 60 nm, (d-f) 30 nm, and (g-i) 10 nm.

15 List of measured gold nanoparticles

Table S2: List of GNPs used in the experiments.

Nominal diameter	Manufacturer	Batch number	size variation
80 nm	BBI solutions	21040063	$\leq 8\%$
60 nm	BBI solutions	19080123	$\leq 8\%$
40 nm	BBI solutions	21040116	$\leq 8\%$
30 nm	BBI solutions	21070139	$\leq 8\%$
10 nm	BBI solutions	21050067	$\leq 10\%$

References

1. Gibson, S. F.; Lanni, F. Experimental test of an analytical model of aberration in an oil-immersion objective lens used in three-dimensional light microscopy. *J. Opt. Soc. Am. A* **1992**, *9*, 154–166.

2. Taylor, R. W.; Mahmoodabadi, R. G.; Rauschenberger, V.; Giessl, A.; Schambony, A.; Sandoghdar, V. Interferometric scattering microscopy reveals microsecond nanoscopic protein motion on a live cell membrane. *Nat. Photonics* **2019**, *13*, 480–487.

3. Mahmoodabadi, R. G.; Taylor, R. W.; Kaller, M.; Spindler, S.; Mazaheri, M.; Kasaian, K.; Sandoghdar, V. Point spread function in interferometric scattering microscopy (iSCAT). Part I: aberrations in defocusing and axial localization. *Opt. Express* **2020**, *28*, 25969–25988.

4. Gonzalez, R. C.; Woods, R. E.; Eddins, S. L. *Digital image processing using MATLAB*; Pearson Prentice Hall: New Jersey, 2004.

5. Trefethen, L. N.; Bau, D. *Numerical linear algebra*; SIAM, 2022; Vol. 181.

6. Dijkstra, E. W. A note on two problems in connexion with graphs. *Numer. Math.* **1959**, *1*, 269–271.

7. Michalet, X. Mean square displacement analysis of single-particle trajectories with localization error: Brownian motion in an isotropic medium. *Phys. Rev. E* **2010**, *82*, 041914.

8. Kashkanova, A. D.; Blessing, M.; Gemeinhardt, A.; Soulat, D.; Sandoghdar, V. Precision size and refractive index analysis of weakly scattering nanoparticles in polydispersions. *Nat. Methods* **2022**, *19*, 586–593.
